# Rotational Dynamics of The Transmembrane Domains Play an Important Role in Peptide Dynamics of Viral Fusion and Ion Channel Forming Proteins—A Molecular Dynamics Simulation Study

**DOI:** 10.3390/v14040699

**Published:** 2022-03-28

**Authors:** Chia-Wen Wang, Wolfgang B. Fischer

**Affiliations:** Institute of Biophotonics, College of Biomedical Science and Engineering, National Yang Ming Chiao Tung University, Taipei 112304, Taiwan; chiawen.wang@gmail.com

**Keywords:** viral fusion and channel proteins, peptide dynamics, transmembrane domains, diffusion coefficient, rotational dynamics, molecular dynamics simulations

## Abstract

Focusing on the transmembrane domains (TMDs) of viral fusion and channel-forming proteins (VCPs), experimentally available and newly generated peptides in an ideal conformation of the S and E proteins of severe acute respiratory syndrome coronavirus type 2 (SARS-CoV-2) and SARS-CoV, gp41 and Vpu, both of human immunodeficiency virus type 1 (HIV-1), haemagglutinin and M2 of influenza A, as well as gB of herpes simplex virus (HSV), are embedded in a fully hydrated lipid bilayer and used in multi-nanosecond molecular dynamics simulations. It is aimed to identify differences in the dynamics of the individual TMDs of the two types of viral membrane proteins. The assumption is made that the dynamics of the individual TMDs are decoupled from their extra-membrane domains, and that the mechanics of the TMDs are distinct from each other due to the different mechanism of function of the two types of proteins. The diffusivity coefficient (DC) of the translational and rotational diffusion is decreased in the oligomeric state of the TMDs compared to those values when calculated from simulations in their monomeric state. When comparing the calculations for two different lengths of the TMD, a longer full peptide and a shorter purely TMD stretch, (i) the difference of the calculated DCs begins to level out when the difference exceeds approximately 15 amino acids per peptide chain, and (ii) the channel protein rotational DC is the most affected diffusion parameter. The rotational dynamics of the individual amino acids within the middle section of the TMDs of the fusion peptides remain high upon oligomerization, but decrease for the channel peptides, with an increasing number of monomers forming the oligomeric state, suggesting an entropic penalty on oligomerization for the latter.

## 1. Introduction

Viral fusion and ion channel proteins adopt a quaternary structure for proper functioning [1,2]. Structural data can be achieved for many of these proteins, even when solely using their transmembrane domains (TMDs), which, in all the cases identified to date, have adopted helical motifs. During the ‘manufacturing’ of the viral membrane proteins, they are thought to exist as monomeric units, eventually structurally relaxed, followed by assembly into their quaternary structures. Macroscopic dynamics, expressed by the diffusivity of the proteins, as well as internal microscopic dynamics, are important to achieve the proper fold, the assembly, and ultimately, the functional state.

All known fusion proteins are bitopic and form trimeric assemblies with helical motifs of their TMDs [1]. It is envisioned that the assembly of these proteins should be rather loose in order to allow for the complex mechanical features that they impose on the lipid membrane during the fusion process. In an analogy of their human companions, the viral channel forming proteins (VCPs) should instead adopt a confined structure for proper functioning, which enables ion and proton flux across the viral or cellular membranes. As for the VCPs [2], for many of them, an answer to how much they comprise an actual assembly and for how long is still lacking [3], since selectivity for most of them is either low or not well defined. In addition, channel properties are suggested to be formed by the assembly of different numbers of aggregated monomers [4,5,6], which explains the high fluctuation of conductance levels observed in the experiments [3,7,8,9].

In this study, a comparison of the TMDs of fusion and VCPs of the same type of virus is made, including structures for which experimental structures are available, and those from pure computational modeling: spike protein (S protein) [10] and E protein of severe acute respiratory syndrome coronavirus type 2 (SARS-CoV-2) and SARS-CoV, gp41 [11] and Vpu, including some computational models, from human immunodeficiency virus type 1 (HIV-1), haemagglutinin [12], and M2 of influenza A [13]. In addition, the fusion protein gB of herpes simplex virus type 1 (HSV-1) [14] is used.

The event of fusion requires several distinct steps, including recognizing a receptor protein of the host that initiates conformational changes of the fusion protein. This process then overcomes the kinetic barrier imposed upon the fusion process by the lipid bilayer [15]. The consequence of the initialization is the approach of the two bilayers, that of the host cell and the viral bilayer, to form a stalk-like hemifusion intermediate state. Overcoming another kinetic barrier, the two bilayers finally disembogue into the pore and thus finalize the fusion process by entering the so-called post-fusion state. During the fusion process, the dynamics of the TMDs play a significant role in promoting and following the lipid membrane dynamics to complete the fusion process [16]. Regarding the role of the TMDs, an important aspect of addressing is that the spatial contribution of the TMDs to the overall size of the fusion protein is minor, and that the association of the extra-membrane units takes the overall responsibility to trimerize. Connected via the linker region to the TMDs, the question arises as to how much the TMDs keep their trimeric assembly during the mode of action, and show essential motions such as the cross-sliding of the TMDs. Or, can they even associate–dissociate during this process [16]? In many cases, it could be demonstrated that distancing the trimers from each other, which are usually held together in GXXXG [17], AXXXG [18] and leucine-isoleucine zipper motifs [19], has an enormous impact on the fusogenicity of the overall protein. Moreover, how much does entropy contribute to this scenario and potentially keep them in a monomer–oligomer equilibrium in the pre-fusion state (e.g., S protein [20])?

Viruses also encode relatively short, mostly around 100 amino acids in length, biotopic membrane proteins identified experimentally to enable ion flux across lipid membranes [2,21,22]. Among the earliest known channel proteins are M2 of influenza A [23,24] and Vpu [25,26]. In recent years, other viruses have been identified to harbor this type of protein. Some viruses encode polytopic proteins, such as p7 of HCV with 2 TMDs and E5 and 3a proteins from HPV and SARS, with 3 TMDs. Structural information is available for many of them. Their function within the infectivity cycle of the viruses can enable leakage-like currents across the lipid membrane [21]. Still, in many cases, the precise functioning remains to be elucidated. All channels to date show a helical motif for their TMDs, making the notation of being like porins questionable [27]. For some of them, like M2, only TMD structures are available, whereas the extra-membrane domains remain to be identified.

To date, M2 is among the best-characterized channels regarding its function as a channel [28]. Detailed mechanical features are proposed, especially for M2 [29]. From molecular dynamics (MD) simulations, it is suggested that the dynamics of the backbone and side chains are uncorrelated [30]. In most cases, the oligomeric state of the proteins that they adopt for proper functioning is well characterized, and manifested in documented experimental structural data. In some cases, like Vpu, a large oligomer with functional subunits is also proposed [6]. Defined conductance states are proposed only for some of them. This finding suggests that they could also be considered as pore-forming proteins [3], and their mechanism of function is not considered to be relatively high-precision functioning.

The diffusion coefficient (DC) is a crucial value that provides macroscopic information about the dynamics of the proteins or peptides by fluorescence correlation spectroscopy, fluorescence recovery after photobleaching, and single particle tracking, to mention some of the methods [31,32]. Some of the measurements are done with complete proteins and transmembrane peptides using giant unilamellar vesicles [31]. Obtaining the DC from structure-based MD simulations have been developed and applied on membrane proteins [33,34,35,36,37] and globular proteins [38,39,40,41].

A series of fusion proteins and VCPs are investigated to identify differences in macroscopic (e.g., diffusivity) and microscopic features (%-helicity and rotational displacement per residue) of these proteins, monitoring their TMDs monomeric or oligomeric state using MD simulations. The data are interpreted in terms of the consequences upon forming a quaternary structure (assembly process of these TMDs), and the relation between the quaternary conformation and the mechanism of function. Experimentally derived TMD structures are compared with the ideal helices of the respective TMDs.

## 2. Materials and Methods

### 2.1. Sequences Used for MD Simulations and Preparation of Proteins

MD simulations have been performed with peptides based on the TMDs of viral fusion proteins and VCPs derived from experimental sources, or based on sequences from the protein sequence data bank as follows:

- S protein of SARS-CoV-2 (PDB ID: 7LC8 [10]), named S:
  ➢ WLGFIAG LIAIVLVTIL LSSTTSC
- haemagglutinin of influenza A virus (PDB ID: 6HJQ [12]), named HA:
  ➢ MGVYQILAIY STVASSLVLL VSLGAISFWM without parts of the linker at the N terminal side (175–184, GVKLESMGVY),
- glycoprotein 41 of HIV-1 (PDB ID: 5JYN [11]), named gp41:
  ➢ NWLWYIRIFI IIVGSILGLR IVFAVLSLVN RVRQGYSPLS
- glycoprotein B of HSV-1 (PDB ID: 5V2S [14]), named gB:
  ➢ GVSSFMSNPF GALAVGLLVL AGLAAAFFAF RYVMRLQSNP
- E protein of SARS-CoV (PDB ID: 5X29 [42]), named E:
  ➢ ETGTLIVNSV LLFLAFVVFL LVTLAILTAL RL
  and in its extended form, named E_58_:
  ➢ ETGTLIVNSV LLFLAFVVFL LVTLAILTAL RLAAYAANIV NVSLVKPTVY VY SRVKNL
- M2 of influenza A virus (PDB ID: 2L0J [13]), named M2:
  ➢ SSDPLVVAAS IIGILHLILW ILDRLFFK
- experimental structure of Vpu of HIV-1 (PDB ID: 1PI7 [43]), Vpu, as well as two different lengths of Vpu (UniProt ID: P05919), named Vpu*_32_ and Vpu*_53_ as ideal helices, depending on the number of amino acids (32 or 53):
   Vpu
    ➢ AIVALVVAII IAIVVWSIV
   Vpu*_32_
    ➢ MQPIPIVAIV ALVVAIIIAI VVWSIVIIEY RK
   Vpu*_53_
    ➢ MQPIPIVAIV ALVVAIIIAI VVWSIVIIEY RKILRQRKID RLIDRLIERA EDS

The following protocols are used to generate the specific helices:

(i) a short fragment (204–210) is added to the C-terminal side of both the experimental and ideal TMD of HA as a short ideal TMD by using Molecular Operating Environment (MOE) software (v2018, Chemical Computing Group, Tokyo, Japan); (ii) a short N-terminal fragment (771–774) was added by using the program LOOPY [44,45] to connect the membrane-proximal external region (MPER) and the TMD of gB; (iii) an extended version of Vpu, Vpu*_53_, was generated as a single long helix, and consequently bent by molecular visualization software, Swiss PDB Viewer 4.1 (http://www.expasy.org/spdbv/, accessed on 18 March 2021 [46]), to position the second helix, since it is found to lie on the membrane surface [47]. The bending was done so that D40 was pointing at the lipid membrane and R49 in the aqueous phase [47]. Finally, a structure was obtained with S24 of the TMD not pointing underneath the newly created surface helix [48]. (vi) A gp41 mutant was generated by exchanging the R696 to L696 using MOE software, and is named R696L. (v) Oligomeric experimental TMDs are used as templates to generate the respective oligomers by superposing the Cα atoms of helixes with backbone dihedrals angles of ϕ = −65°, and ψ = −39° to experimental structures using MOE software. These models are referred to as ‘ideal’ structures. The structures were consequently energy-minimized, including the steepest descent, followed by conjugated gradient and truncated Newton calculations. The ideal helices from the experimental structures are named in the text. The newly generated ideal structures are indicated by a star in the superscript. The trimeric conformations of M2, E, and all artificial Vpu*_32_ oligomers ^5^Vpu*_32_, were created by using in-house software Prediction of Ion Channel Assembly (PICA) (see below). Oligomers of the TMDs, marked with a superscript number, indicating the number of TMDs in their oligomeric state.

### 2.2. MD Simulations

The experimental and ideal structures, both monomers and oligomers, were transformed into the united atom model by using program package GROMACS version 4.6.7 (University of Groningen, Groningen, The Netherlands) for gB, gp41 and M2, and version GROMACS 2019 (University of Groningen, Groningen, The Netherlands) for S, E, HA and Vpu, applying the Gromos96 ffG45a3 force field (ff). The transformed peptides were embedded into the 1-palmitory-2-oleoyl-*sn*-glycero-3-phospho-chloine (POPC) phospholipid bilayer patch, which was obtained from an external source (P. Tieleman, University of Calgary, Calgary, Alb., Canada, former download site), by using the Gromacs option command *membed* [49,50]. The protein was inserted as a string along the *z*-axis into the lipid membrane, and surrounded by a spatial buffer region around the protein with a radius of 0.22 nm. The protein was then gradually expanded to its original size and shape. Lipid molecules overlapping with the protein were deleted during this process. Each system contains 288 POPC lipid molecules (144 molecules per leaflet), with about 8750 to 17,500 simple point charge (SPC) water molecules before protein insertion.

Due to the asymmetric conformation between the N and C terminal of E_58_ and Vpu*_53_, the insertion of the peptide by command membed will cause an uneven number of lipids to be removed from each leaflet. Therefore, the simulation box was enlarged to host a second peptide, which was reversed inserted into the lipid bilayer to compensate for the uneven removal of the lipid molecules when one protein is inserted, and consequently generating an equal number of lipid molecules to be removed from each leaflet. The enlarged box contained 512 POPC lipid molecules (256 molecules per leaflet) and 58,470 SPC water molecules.

An adequate amount of counter ions (Cl^-^) was added to the system to compensate for additional charges of the proteins. The system was equilibrated using the steepest descent and conjugate gradient. Due to the insertion, the gaps between protein and lipid molecules were eliminated by gradually increasing the temperature from 0 to 310 K, combined with releasing restraints on the protein from 1000 to 0 kJ mol^−1^ nm^−2^. The total equilibration time was about 1.6 to 1.9 ns. The production run was conducted under NPT condition for 500 ns, with 2 fs time step length for all samples. The temperature was coupled at 310 K by Nose–Hoover coupling with a coupling time of 0.1 ps. The pressure was coupled using Berendsen semi-isotropic coupling (*x*-*y* directions) at 1 bar with a coupling time of 2 ps.

### 2.3. Diffusion Coefficient (DC)

The translational diffusion coefficients of proteins in lipid membrane under the periodic boundary condition simulation box (D_TPBC_) were calculated by an online calculator (https://diffusion.lobos.nih.gov/, accessed on 6 July 2020 [51,52]) using the following equation, developed based on the Saffman–Delbrück model:

(1) $$D_{\mathrm{TPBC}}=\frac{k_{B}T}{2L^{2}}\sum_{k\neq 0}\frac{1}{\eta_{m}k^{2}+2\eta_{f}k\;\tan h\left(\mathrm{kH}\right)}e^{-k^{2}{\left(R/L_{\mathrm{SD}}\right)}^{2}R^{2}/2}$$

with k_B_ = Boltzmann’s constant, T = temperature, L = length of the lipid membrane, η_m_ = membrane viscosity, η_f_ = surrounding fluid viscosity, k = wave factor, h = thickness of lipid membrane, H = one-side fluid height of the simulation box, R = radius of gyration of the protein, and L_SD_ = Saffman–Delbrück length (L_SD_= η_m_/2η_f_).

The following corrected equation was used to obtain the translational diffusion coefficient in an infinite environment (D_T0_) [38,39]. In the equation, ξ(H^*^) = ln [1 + (πH/2L_SD_)] + e − 1.

(2) DTPBC≈DT0+kBT4πηm(lnLLSD)−ξ(H*)(1+H/LSD)

The rotational diffusion coefficient for a membrane protein under periodic boundary condition (D_RPBC_) [53] was calculated using

(3) $$D_{\mathrm{RPBC}}=\frac{k_{B}T}{4{\pi \eta }_{m}R^{2}}\left(1-\frac{{\pi R}^{2}}{A}\right)$$

where A is the area of the simulation box.

The size correction of the rotational diffusion coefficient in an infinite system (D_R0_) was done according to [53],

(4) $$D_{\mathrm{RPBC}}=D_{R0}-\frac{k_{B}T}{4\eta_{m}A}$$

Error calculations were done as described in the literature [38,39,52,53,54].

All calculations were done using the patch size of 288 lipid molecules as the standard system. According to the equations, the analysis of the DCs is box size-dependent, and as a result, the analysis of the larger lipid patches for E_58_ and Vpu*_53_ consisting of 512 lipid molecules was using values from the E and Vpu*_32_ with the same protein oligomerization state and lipid patch size as 288 lipid molecules.

### 2.4. Generation of Artificial Oligomeric Structures

In-house software, Prediction of Ion Channel Assembly (PICA), was used to generate the artificial oligomeric conformations from experimental or ideal monomeric TMDs. The TMDs were positioned along a central axis and simultaneously moved to screen the conformational space of the oligomers. The search space included distance (in steps of 0.1 nm, covering a total range of 0.8–18 nm, measured as the distance of the center of mass of the TMDs), tilt (in degrees of ±4° to a maximum of ±40°), and rotational angles (in degrees of ±10° of a total of ±60°), which used F26, H37, and S24, pointing to the pore center as 0° for E, M2, and Vpu*_32_, respectively. All steps were applied simultaneously to all the TMDs. After positioning, the structures were operated through a short minimization (steepest descent), followed by calculating the potential energies of the structures using the g_energy command and Gromos 96 45a3 ff of the Gromacs suit. All generated structures were ranked according to the potential energy, and the best ranked structures were used for following MD simulations. In the case of ^5^Vpu*_32_, a bundle ranked as 26th was also used for data generation and analysis (^5^Vpu*_32−r26_).

### 2.5. Data Analysis

The radius of the gyration of each of the peptides was calculated by using (i) the entire peptide (long peptide protocol, lpp) and (ii) the stretch of just 23 amino acids (short peptide protocol, spp). A sequence of 21/19 amino acids were used for the S/Vpu peptide for both protocols, in as much as the experimental structure contains only this number of residues.

The calculation of the residue rotation degree per ns was done using the data from trajectories covering 100–500 ns. In contrast, the entire length of the trajectory was used to calculate the other parameters.

An arc was fitted to the data of the standard deviation (STD) of rotational dynamics of the Cα atoms of the amino acids, to quantify the shape of the data pattern using a non-linear implicit circle fitting function in Origin9 data analysis and graphing software (OriginLab, Northampton, MA, USA) in its default mode. The stretch of amino acids within the TMD for which their respective STD values were used was chosen by visual inspection.

### 2.6. Hard and Software

The MD simulation set up and equilibration run were generated on a Dell Precision 5820 Tower workstation with 4 Intel Xeon W-2125 4.0 GHz CPU cores. The equilibrated systems were submitted to the National Center for High-performance Computing (NCHC), Hsinchu, Taiwan, and the production run finished on the supercomputer Taiwania, using 64 Intel Xeon Gold 6148 2.4 GHz CPU cores. The molecular visualization software used in the study was MOE and Visual Molecular Dynamics (VMD) (v1.9.2, University of Illinois, Urbana-Champaign, Urbana, IL, USA). Plots were made using Origin9 data analysis and graphing software. The program Protter-v1.0 (http://wlab.ethz.ch/protter/start/, accessed on 4 November 2019–18 November 2021) [55] was applied to visualize the position of the amino acids with respect to the lipid bilayer.

## 3. Results

**Figure 1:**
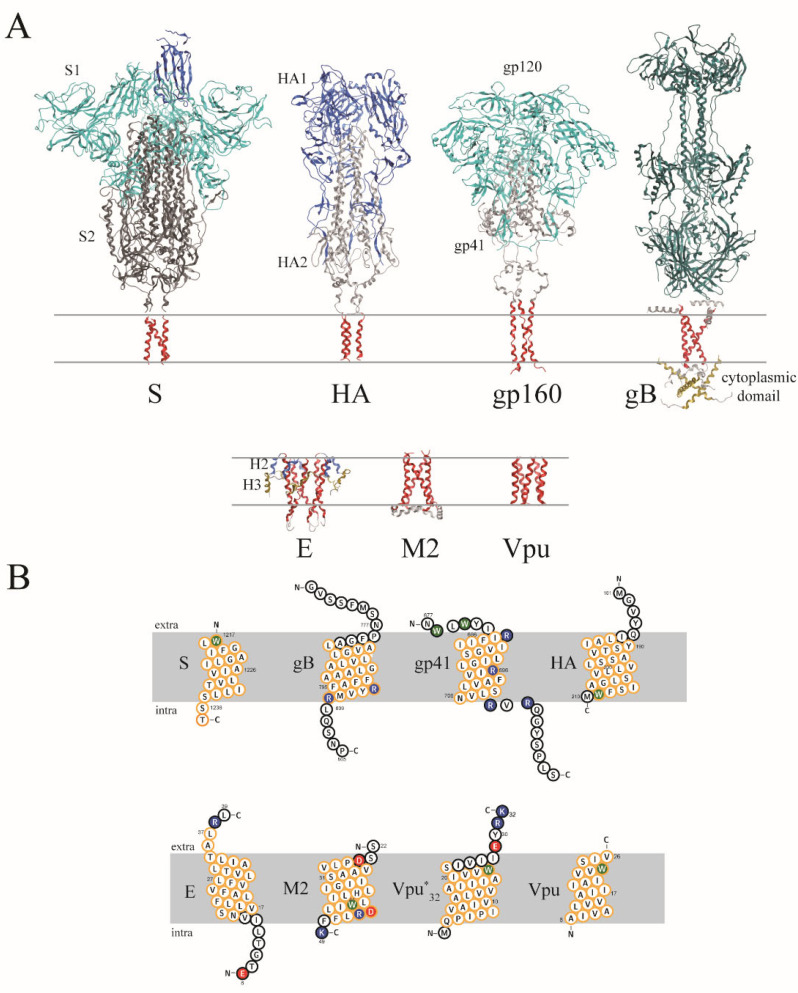
(**A**) Models in their backbone representation of fusion proteins based on experimental data for S protein of S1 and parts of S2 (combined PDB ID: 6VSB and PDB ID: 7LC8 (TMD) for visualization only), haemagglutinin (PDB ID: 6HJQ), gB (PDB ID: 5V2S) and gp160 (combined use of PDB ID: 6PWU (gp120 and parts of gp41), and PDB ID: 6E8W (linker and transmembrane domain of gp41) for visualization only), as well as the viral channel forming proteins (VCPs) M2 (PDB ID: 2L0J), E protein (PDB ID: 5X29) and Vpu (PDB ID 1PI7). The TMDs are shown in red, while the extra-membrane domains are shown in different colors, highlighting the different subdomains. Different subunits of the proteins are marked as S1/2 or HA1/2. The lipid membrane is approximated by two lines. (**B**) The sequence and topology of the fusion proteins (upper row) and VCPs (lower row), which are used in the simulations, are shown using the program ‘Protter’. The residues assumed to be within the lipid membrane are embedded within the grey bar. Residues encircled in orange are residues chosen for the calculation of the diffusion coefficient using spp. Positively charged residues are shown in blue; the negatively charged residues in red. Tryptophan residues are highlighted in green. Extra-membrane residues are manually reorganized in the plots.

The transmembrane domains of a series of fusion proteins and VCPs, for which structural information is available, are used to analyze the dynamics of their TMDs (Figure 1A). The structural motif of the TMDs from experiments is a helix. Those residues, identified as being embedded within the lipid membrane, vary in their amino acid sequences (Figure 1B). The stretch of the TMD within the protein’s amino acid sequence is supported by using a series of secondary structure prediction programs (Supplementary Figure S1). The amino acids at the flanks of the TMDs vary from hydrophobic, e.g., W—containing, in S, HA, Vpu, to W and positively charged residues—containing, e.g., as in gp41, gB and M2. TMDs of the ion channel protein M2 and Vpu are the only TMDs—containing negatively charged residues close to the core of the hydrophobic stretch of their TMDs.

### 3.1. Helicity

**Figure 2:**
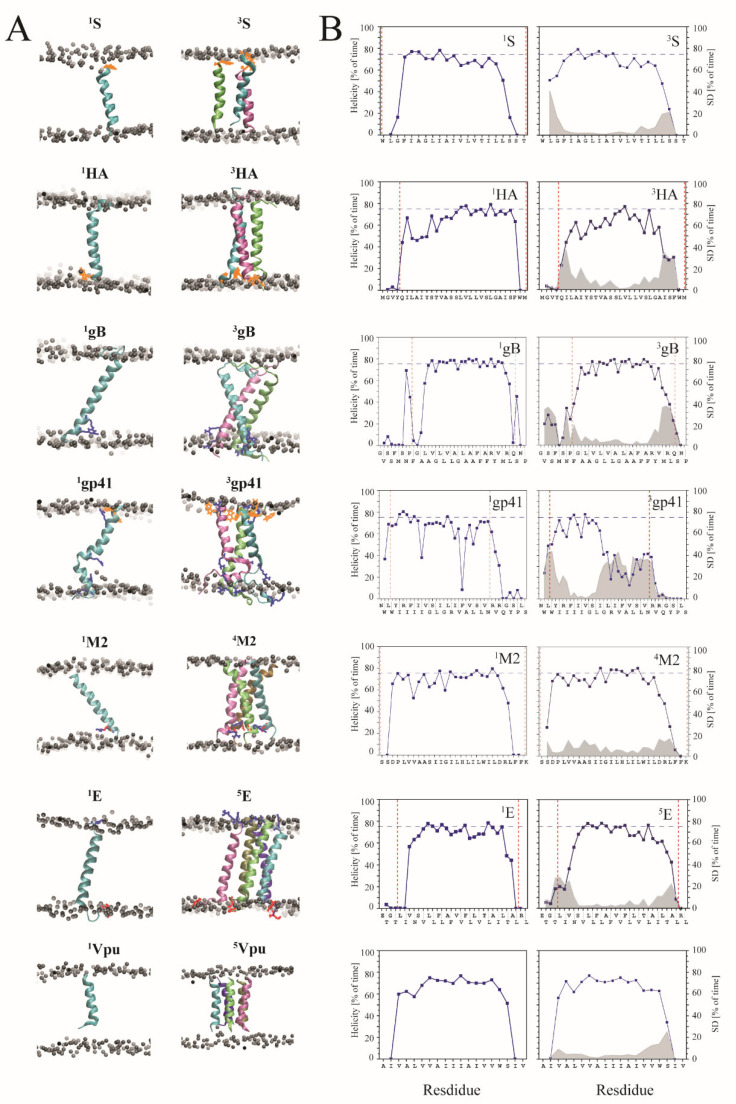
(**A**) Structural models of the peptides in the monomeric and oligomeric state at the end of the 500 ns MD simulations of S protein (^1^S, ^3^S), HA (^1^HA, ^3^HA), gB (^1^gB, ^3^gB), gp41 (^1^gp41, ^3^gp41), M2 (^1^M2, ^4^M2), E peptide (^1^E, ^5^E), and Vpu peptide (^1^Vpu, ^5^Vpu). The structures were taken from experimentally available data. The boundaries of the lipid membrane are marked by grey spheres representing the phosphorous atoms of the lipid head groups. The remaining lipid atoms as well as the water molecules are omitted for clarity. (**B**) The corresponding percentage of time of each residue being in a helical conformation is shown for both monomer (left column) and oligomer with standard deviation in grey when averaging the values for each individual TMD (right column). Red dash lines indicate the boundaries of the lipid membrane.

During 500 ns MD simulations of the individual TMDs embedded in hydrated lipid bilayers, the TMDs adopt a stable structure, indicated by the leveling of the values of the root mean square deviation (RMSD) of the structure files (Supplementary Figure S2). The percentage of time for the TMDs of fusion and channel peptides remain in a standard helical conformation, provided that the hydrogen bonds and φ/ψ angles, referred to as helicity, are on a 75% level for most of the core residues within the TMD. This result is also independent of whether the TMDs are simulated in their monomeric or oligomeric state (Figure 2). Exceptions are found for HA towards its N terminal side, and peptide ^1^gp41, which shows a substantial loss of helicity from R696 to S703. In the trimeric state, ^3^gp41, the three helices unwind even more from I693 towards their C terminal side.

The same data trend is observed when analyzing the ideal peptides (Supplementary Figure S3A–D). An exception is gp41, which in both ^1^gp41 and ^3^gp41 forms, retains helicity compared to the experimental peptides (Supplementary Figure S3A,B). In the case of the mutant R696L, both in its experimental monomeric and oligomeric state, a strong loss of helicity is observed from L696 towards the TMD core region from the C terminal side (Supplementary Figure S3E-I,F-I), but no unwinding in the trimeric form. The same pattern also appears in ideal trimeric R696L. However, the helicity of oligomeric R696L is less dynamic than oligomeric gp41. The %-helicity of the residues from the TMD region when simulating an extended E peptide, ^1^E_58-a/b_ and ^3^E_58-a/b_, does not change the pattern compared to the data for ^1^E and ^3^E (Supplementary Figure S3E-II,F-II). Artificial trimer, ^3^E, delivers the same pattern of a stable helical core region, whereas the helicity of ^3^M2 decreases (Supplementary Figure S3F). Furthermore, during the simulation of Vpu*_32_ and Vpu*_53_, independent of their oligomeric state, these structures lose helicity of W23 and the amino acids towards the C terminal side (Supplementary Figure S3G,H).

In both cases: a hydrophobic residue that is near the same lipid headgroup region as HA, and charged residues within the core region of the TMD as in gp41, leads to reduced %-helicity for some turns of the helix. Charged residues or tryptophans do not necessarily lead to a high percentage of unwinding; it is the oligomeric state of the peptide which amplifies the trends.

### 3.2. Translational and Rotational DCs

**Figure 3:**
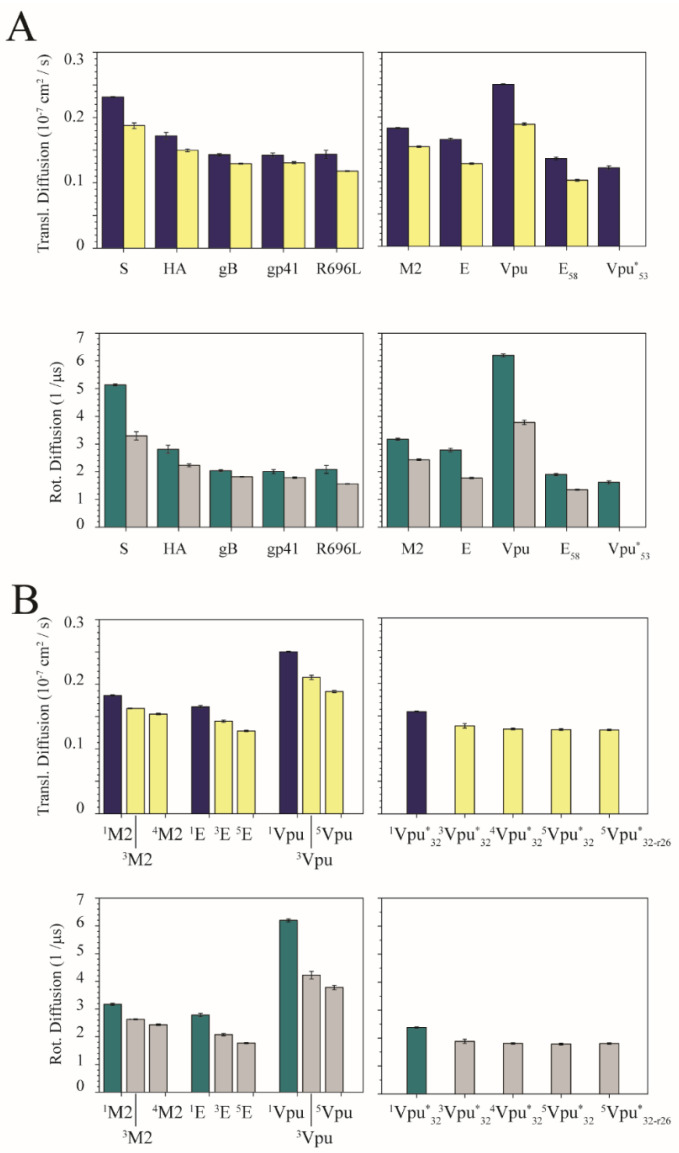
(**A**) Translational (upper panel) and rotational diffusion coefficients (DCs) (lower panel) of fusion (left column) and channel peptides (right column) from MD simulations of the experimental structures. The data are calculated using lpp. The blue and green bars represent the data for the simulations of the monomers, while the yellow and grey bars represent the data of the oligomers (3 TMDs for each fusion protein; 4 TMDs for M2, and 5 TMDs for E and Vpu proteins). The subscripts for Vpu and E protein mark the number of amino acids used in the simulation. The star in Vpu*_53_ indicates that this structure is derived from an ideal helix (see Materials and Methods). (**B**) DCs for specific oligomeric structures using the same calculation (lpp) and color scheme as above. Left column shows the DCs of experimental ion channel peptides in monomeric and tetra-/pentameric states together with trimeric conformations, which are done by using the in-house software Prediction of Ion Channel Assemblies (PICA), by taking experimental monomers as building blocks for the assembly. Right column shows the PICA-generated oligomeric bundles by using the ideal monomeric structure of Vpu. The superscripts mark the oligomeric state which was used in the simulations. For ^5^Vpu*_32_, the tryptophan residues are pointing inside the pore, while r26 marks the respective pentameric bundle, ^5^Vpu*_32-r26_, which the tryptophan residues are pointing to outside the bundle leaving the S24 pointing inside the putative pore. This kind of orientation also appears in the trimeric and tetrameric bundles.

Dependent on the oligomeric state, the calculated translational DCs of the TMDs using lpp are larger when the TMDs are simulated as monomers compared to the calculated values for the oligomers (Figure 3A). The DCs of the fusion peptides follow the sequence S > HA > gB ~ gp41 ~ R696L. The DCs of the ion channels follow Vpu >> M2 > E > E_58_ > Vpu*_53_. The DC of the Vpu peptide is the largest, compared to the DCs of the fusion proteins. This result is due to the length of the peptide, which leads to a smaller radius of gyration of Vpu. An increasing number of oligomers, see M2, E and Vpu, also leads to a decrease in both rotational and translational DCs (Figure 3B). In the case of various Vpu*_32_ oligomers, changes of the DCs are marginal.

Calculating the DCs using spp still shows larger values for the monomers than the oligomers. (Supplementary Figure S4A,B). The decrease of the DCs for the fusion peptides when going from the monomeric state to the trimeric state is around 15% for the translational DCs, and close to twice as large for the rotational DCs (Supplementary Table S1). This is independent of the protocol used for the calculations. In the case of the channel peptides, which adopt oligomers with four or more TMDs, the difference is even larger than that for the fusion peptides in the spp protocol. The values are around 25% for the translational DCs and 40% for the rotational DCs. Calculations with artificial trimeric channel oligomers reveal that the differences are barely discriminable from those values for trimeric fusion peptides. The DCs decrease gradually with an increasing number of oligomers, which is the result of increasing the radius of the gyration of the protein (Supplementary Figure S4B for M2, E and Vpu and Supplementary Table S1).

**Figure 4:**
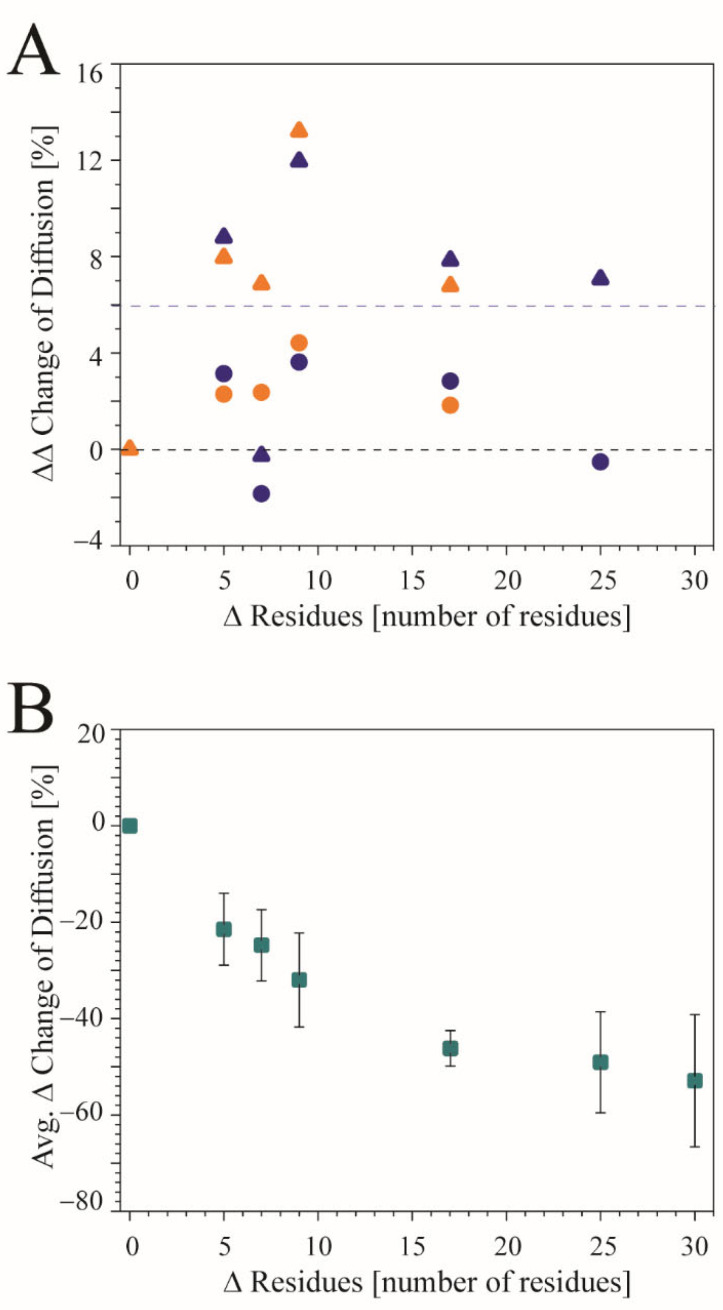
(**A**) The percentage change (ΔΔ change) of the diffusion coefficients (DCs) between spp and lpp between monomeric and oligomeric states using the monomeric state as reference [(oligomeric state (lpp–spp))–(monomeric state (lpp–spp))]. The ΔΔ changes of DCs that are bigger than zero indicate that the decrease is larger between spp and lpp in the monomeric state than in the oligomeric state. Numbers smaller than zero indicate that the DCs decrease more between spp and lpp in the oligomeric state. The data do not discriminate between fusion and ion channel proteins. The blue and orange circles refer to the translational DCs taken from simulations of the experimental and ideal structures, respectively. The triangles refer to the respective rotational DCs (blue for experimental structures and orange for ideal structures). (**B**) Data of the differences in the DCs from the same amino acid number difference, Δ Residues, between two protocols, (lpp–spp) are averaged over all translational and rotational DCs independent of the type of peptide and oligomeric state.

The percentage change between spp and lpp of all the diffusion coefficients (DCs) between monomeric and oligomeric states shows that the changes for the rotational DCs are more significant than those for the translational DCs. This is the case independent of whether experimental or ideal TMDs are used (Figure 4A). Averaging DC percentage change between two protocols based on the residue-length difference, independent of fusion and channel TMDs, and monomers or oligomers, reveals a decline in the values, with increasing difference in the numbers of the amino acids (Figure 4B and Supplementary Table S2). At a difference of approximately 15 amino acids per peptide chain, the values level off.

Based on *p*-values, most of the lpp values are different from each other (Supplementary Table S3). Thus, the lpp data primarily reflect the dynamics of the peptides. Numbers calculated according to the spp, referring to the TMD core region internal dynamics from sequence difference, are more likely be less discriminable. Ideal helices follow the trend described for the experimental TMDs.

### 3.3. Rotational Dynamics

**Figure 5:**
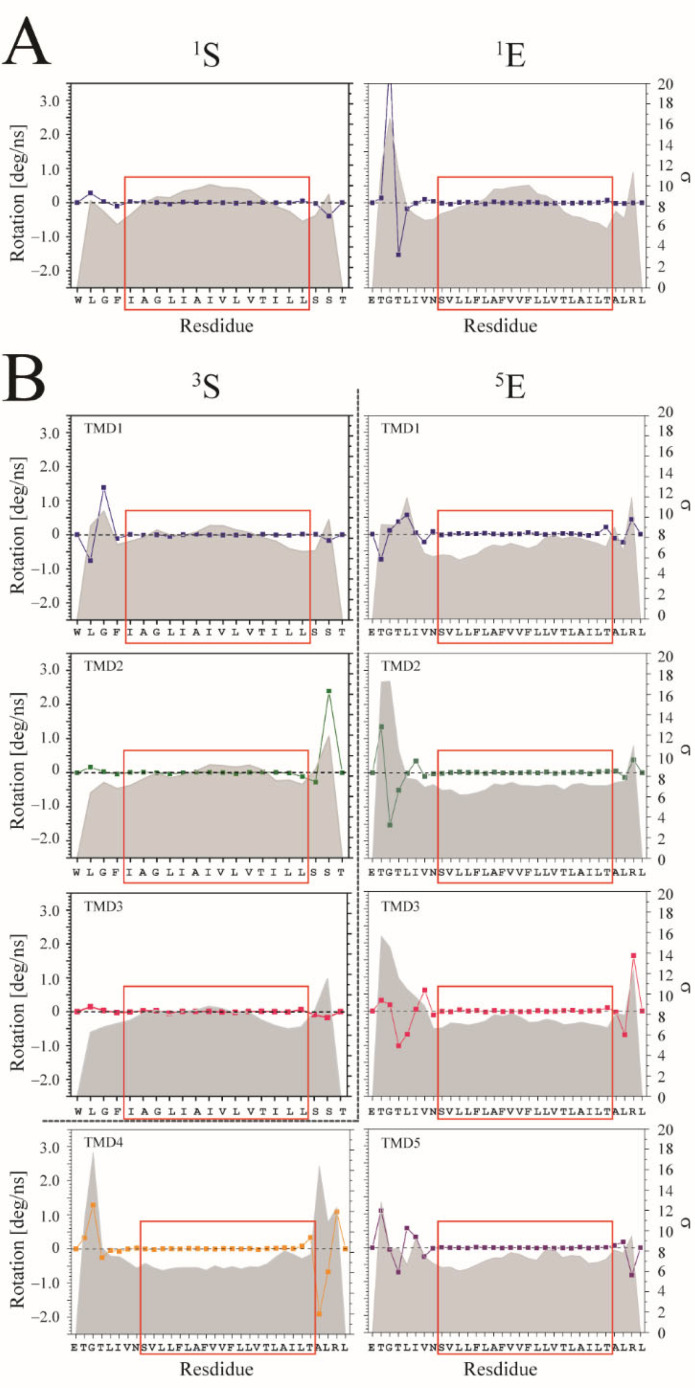
Averaged degree of rotation of the Cα atoms per time step (ns) during the MD simulation for each of the residues of (**A**) the experimental monomeric peptides S and E, as well as (**B**) for their individual TMDs when they are simulated in their respective experimental oligomeric states. The standard deviation (STD) is shown as a grey line. The values are calculated from simulations of the experimental structures. The red boxes mark the amino acids used for calculating the curvature from the respective values of the STD. The superscript indicates the oligomerization state of the protein.

The degree of rotation per ns is calculated for each Cα-atom of the TMDs and averaged over the entire trajectory. In comparison to the residues located towards either end of the TMDs for both the S and E proteins, the residues within the center of the TMDs fluctuate the most when simulating the TMDs as monomers (Figure 5A). When plotting the values of the STD, the values follow a *w*-pattern, which has large values at the termini as well as in the middle of the TMD. In the trimeric state of the S protein, the *w*-pattern for the individual TMDs remains, but disappears when plotting the standard deviation data for the individual TMDs from simulations of the pentameric E protein (Figure 5B). A similar *w*-pattern is also observed for the monomeric TMDs of the fusion peptides HA, gp41, R696L, and gB, and the channel peptides M2 and Vpu TMDs (Supplementary Figure S5). Furthermore, when simulating the ideal monomers and oligomers, the abovementioned feature is observed for the residues in the center of the TMDs with respect to their STD values of the rotation per ns (Supplementary Figure S5).

Radii between 7.6 and 26.3 artificial units for the TMDs of the monomers of the fusion peptides and channel peptides are obtained (Supplementary Table S4), fitting an arc to the central regions of the TMDs (red boxes in Figure 5 and Figure S5). Upon trimerization of the fusion peptides, the changes in radii vary from 2 to 66%, taking the monomeric peptides as reference points.

Average radii are up to 40,000 times larger in the oligomeric state than the monomer for all channel peptides, regardless of the structure used—experimental or ideal (Supplementary Table S4, arrow mark). Only in some of the ^5^E and ^5^Vpu oligomeric structures do the individual TMDs adopt smaller radii. This is due to the collapse of the bundle structure during the simulation (see also [56]), and the consequent isolated specific TMDs that can then be considered monomers. On the other hand, when simulating trimeric forms of the channel peptides, TMDs that adopt radii as small as those for the fusion peptides increase proportionally without conformation collapsing.

In the special case of ^4^Vpu*_32_, all four TMDs reveal small radii, while for ^5^Vpu*_32_ and ^5^Vpu*_r26_, all of their TMDs show large radii (Supplementary Table S4). This pattern indicates that the tetrameric form of Vpu might still behave like a fusion peptide and is also showing the *w*-pattern on the rotational fluctuation (Supplementary Figure S5). At the same time, its larger oligomers, like the pentamers, can adopt properties of the channel peptides.

While the TMDs of the fusion peptides retain their rotational dynamic within the core region of the TMD when in their oligomeric form, this dynamic is lower for the channel peptides upon assembling into oligomers with more than 3 TMDs. It is likely that the dynamics of the central region of the TMDs are affected by the oligomeric state of the peptide, and with this, affecting the mechanism of the function of the protein.

## 4. Discussion

### 4.1. Model Evaluation

MD simulation is used as an analytical tool to identify dynamical and structural features of a series of TMDs of two classes of viral proteins, fusion, and channel proteins. In many experimental studies regarding the determination of structural details, the assembled TMDs cannot be resolved. This sparks the idea that these entities are decoupled in their dynamics from the extra-membrane domains. Based on their mechanism of function, it can be assumed that TMDs of fusion proteins and ion channel proteins should obey different mechanical and dynamic features. In the case of the fusion TMDs, in the pre-fusion state, the TMDs form trimers and obviously need to dissociate to enable the complex dynamics of the fusion process.

In contrast, TMDs of the ion channel should organize in a well-defined oligomeric state to allow a continuous mechanism: the transduction of ions across the lipid membrane. It is found experimentally that the conductance states of the VCPs vary [3,7,8,9]. This variation is explained by the formation of aggregates of different numbers of monomers [4,5,6], such as the mechanism proposed for, e.g., the antimicrobial peptide alamethicin [57,58,59]. Pore formation via assembly could show slower conductance level changes than those changes occurring via faster gating mechanisms, such as conformational changes observed in eukaryotic/prokaryotic ion channels. Since either two forms of conductance patterns are observable for VCPs, none of the mechanisms regarding gating/pore formation can be ruled out. Following an ‘assembly mechanism’, conformational changes between the conformational stages of the monomers upon assembling could be a second step of subtle conformational changes to allow conductance. This is especially true when smaller aggregates are involved, e.g., trimers to pentamers or hexamers. Thus, the observed data allow for the interpretation of the behavior of the TMDs of VCPs when in low aggregation number.

All the simulations have been conducted in the peptides embedded in POPC lipids, since these lipids are the most abundant lipids identified in the organelles of mammalian cells, including the plasma membrane, endoplasmic reticulum, and Golgi apparatus [60,61]. This allows for a remarkably close approximation of the immediate environment of the peptides within the lipid bilayer supplemented by embedding the system into sufficient water molecules.

The most extended structures of the relevant VCPs are used for MD simulations, albeit in some cases, the shorter version of the VCP is available [62].

### 4.2. Data Evaluation

Interpretation of the data is limited to the TMDs in this study. Decoupling from the extra-membrane parts is assumed, since the fusion proteins have linker regions between the TMDs and their extra-membrane parts. These linker regions may, to some extent, detach the dynamic features of the TMDs from the extra-membrane domains to allow for independent dynamics. The VCPs considered in this study are much smaller than the fusion proteins, and even than their eukaryotic/prokaryotic companions. In addition, for many bitopic VCPs, structures of the extra-membrane domains are not resolved. This result suggests that significant dynamics of these parts of the proteins must exist. These dynamics make it difficult for experimental structural techniques to obtain structural information to date.

Calculating the DCs with two different protocols allows the investigation of the contribution of the MPER of the TMDs on their dynamics. The data suggest that larger MPERs, or an increasing number of amino acids beyond the core region, lower the diffusion of the TMDs. Nevertheless, the change of the differences in the DCs, when calculating with two different lengths of the peptides, levels when the difference of peptide length reaches around 15 amino acids of each peptide chain. This finding is independent of the oligomeric state and the type of peptide. When restricting the calculations to the core region of the TMDs using the spp, the diffusion becomes less discriminative, not only within the two classes, but also across these classes. These trends are for the single TMDs, and when in their biological relevant oligomeric state.

The relative rotational diffusivity is larger than the translational diffusivity, based on calculating the relative difference between the DC values of the monomeric and oligomeric state using the two protocols, spp and lpp. The result shows that the oligomeric channel proteins form more stable conformations than the trimeric fusion proteins. This reflects the concept that fusion proteins execute their job through a complex mechanism, which involves more remarkable spatial dynamic changes. In contrast, channel proteins follow more localized mechanisms to achieve their function. The result also indicates that the difference is leveled when amino acids are present in the MPER.

The translational and rotational DCs of the TMD of monomeric Vpu are the largest of the entire data set. This is due to the shorter peptide length, which results in a smaller radius of the gyration of Vpu than for the other peptides. These data explain the fact that experimental structures of the oligomeric Vpu are still lacking.

A detailed amino acid-based analysis uncovers that the rotational dynamics of the individual amino acids of the TMDs show different behavior for fusion and channel proteins. Rotational dynamics of the amino acids in the core region of the TMDs of fusion peptides remain high, even when the TMDs are in an oligomeric state; here, the trimeric state. This indicates that the internal rotational dynamics are higher in the center of the TMD along the vertical direction for fusion proteins, even when they are in the oligomeric state. Thus, the fusion protein oligomeric state represents a ‘looser’ organization of the TMDs, ready to dissociate again, when the complex lipid dynamics of the fusion process require it. The higher rotational dynamics of the core region are not observed for the channel peptides. Consequently, the TMDs of channel peptides should experience an entropic cost upon oligomerization, which must be compensated by enthalpy-driven interactions in the oligomeric state. The entropic costs assume that the energy could be released to allow for, e.g., twist- or rotational-like and tilt/swiveling motions of TMDs, as indicated for the mechanics of the gating of ion channels [63,64,65,66,67,68].

Based on the data from the macroscopic rotation status obtained by DC calculation and the microscopic internal rotation dynamics, they indicate that the channel proteins become less dynamic when forming oligomeric conformations compared to fusion proteins. In contrast, since the artificial trimeric channel proteins lose this feature, and tend to behave more like fusion proteins, there is a general trend of also losing internal rotational dynamics upon larger oligomers.

A common feature that charged amino acids within the lipid membrane is unfavorable for the integrity of the helical motif of a TMD is supported by the MD simulations in the case of gp41. Oligomerization seems to amplify the effect.

In many cases, structural features of TMDs are not available. A helical motif is proposed based on the amino acid sequence most of the time when using secondary structure prediction programs. Consequently, ideal helical TMDs can be generated and used in MD simulations. How much are the data different from those data when using experimental structures? Based on the data discussed in this study, the differences, in most cases, are not very large. Thus, simulations with ‘ideal structures’ are a reasonable route to obtain sensitive information about the TMDs.

## 5. Conclusions

Fusion and channel proteins form conceptually distinct types of oligomers. The oligomers of the former need to stay dynamic, and within the TMDs, to perform the artistic mechanics anticipated during the fusion process, while those of the latter should come together and stay like that to complete the task. Focusing on the TMD core region, the diffusivity, primarily rotational, is more strongly inhibited in most of the channel protein oligomers than in the fusion proteins. Moreover, both translational and rotational diffusivities are affected mainly by the MPER of the TMDs. On the individual amino acid level, the rotational dynamics are suggested to make a difference between the fusion TMDs and the channel TMDs when in their respective functional oligomeric states. The dynamics remain high for the fusion TMDs, and are decreased for the channel TMDs compared with their monomeric form. The decreased or entropic costs can be referred to as a spring-loaded mechanism to trigger the mechanics involved in the gating mechanisms.

## Supplementary Materials

The following supporting information can be downloaded at: https://www.mdpi.com/article/10.3390/v14040699/s1, Table S1: Difference of DCs between monomer and oligomer; Table S2: Difference of DCs between spp and lpp; Table S3: The *p*-values for the pairwise comparison of the DCs; Table S4: Radii of fitted curves to the standard deviation of the rotational dynamics. Figure S1: Prediction of helical motifs in the transmembrane domains; Figure S2: RMSD of peptides used in a 500 ns MD simulation; Figure S3: Structure models after 500 ns MD simulations and the helicity in percentage of time through 500 ns MD simulation; Figure S4: Translation and rotational DCs of peptide samples; Figure S5: Degree of rotation of the Cα atoms per time step (ns) during the MD simulation.
