# Supplementary material for: Rotational Dynamics of The Transmembrane Domains Play an Important Role in Peptide Dynamics of Viral Fusion and Ion Channel Forming Proteins—A Molecular Dynamics Simulation Study
Source: Viruses. 2022 Mar 28;14(4):699. doi: 10.3390/v14040699 (PMC9024552; doi:10.3390/v14040699)
Supplement: Supplementary file 1 [file viruses-14-00699-s001.zip › viruses-1602999-supplementary.pdf]

viruses

MDPI

Supplementary materials

Rotational Dynamics of the Transmembrane Domains Play an Important Role in Peptide Dynamics of Viral Fusion and Ion Channel Forming Proteins – a Molecular Dynamics Simulation Study

Table S1. Difference of diffusion coefficients (DCs) between monomer and oligomer, referring the monomer diffusion coefficient value as 100 %.

Translational diffusion coefficient

**experimental structures**	spp				lpp			
	Δ	Δ%	Ave. Δ%	*p*	Δ	Δ%	Ave. Δ%	*p*
^{3}S	−0.04	−18.9	−15.3 ± 3.6	-	−0.04	−18.9	−13.5 ± 4.9	-
^{3}HA	−0.02	−10.8			−0.02	−12.8		
^{3}gp41	−0.04	−18.3			−0.01	−8.0		
^{3}gB	−0.03	−12.4			−0.01	−9.6		
^{3}R696L	−0.04	−16.0			−0.03	−17.9		
^{5}Vpu	−0.06	−24.5	−23.4 ± 3.2	**	−0.06	−24.5	−21.9 ± 4.2	*
^{4}M2	−0.04	−18.8			−0.03	−15.7		
^{5}E	−0.06	−26.3			−0.04	−22.6		
^{5}E$_{58-a/b}$	−0.05	−24.0			−0.03	−24.7		
^{3}Vpu	−0.04	−15.8	−15.4 ± 0.8	0.952	−0.04	−15.8	−13.4 ± 2.4	0.984
^{3}M2	−0.03	−14.4			−0.02	−10.9		
^{3}E	−0.04	−16.0			−0.02	−13.5		
**(ideal structures**	spp				lpp			
	Δ	Δ%	Ave. Δ%	*p*	Δ	Δ%	Ave. Δ%	*p*
^{3}S	−0.03	−12.7	−15.6 ± 2.2	-	−0.03	−12.7	−13.5 ± 1.8	-
^{3}HA	−0.03	−15.9			−0.02	−13.4		
^{3}gp41	−0.04	−18.2			−0.02	−16.7		
^{3}R696L	−0.04	−16.8			−0.02	−12.3		
^{3}gB	−0.03	−14.2			−0.02	−12.6		
^{5}Vpu	−0.06	−25.9	−24.9 ± 2.9	**	−0.06	−25.9	−22.7 ± 3.2	*
^{4}M2	−0.05	−21.6			−0.04	−19.5		
^{5}E	−0.06	−27.1			−0.04	−22.8		
^{5}Vpu$^{*}_{32}$	−0.05	−22.4			−0.03	−17.5		

Rotational diffusion coefficient

**experimental structures**	spp				lpp			
	Δ	Δ%	Ave. Δ%	*p*	Δ	Δ%	Ave. Δ%	*p*
^{3}S	−1.84 ± 0.07	−35.9	−28.4 ± 7.3	-	−1.84 ± 0.07	−35.9	−23.7 ± 10.5	-
^{3}HA	−0.84 ± 0.04	−20.2			−0.58 ± 0.07	−20.5		

^{3}gp41	−1.68 ± 0.08	−33.9			−0.22 ± 0.04	−10.9		
^{3}gB	−0.95 ± 0.02	−21.1			−0.22 ± 0.02	−10.7		
^{3}R696L	−1.54 ± 0.09	−30.7			−0.52 ± 0.06	−24.9		
^{5}Vpu	−2.42 ± 0.04	−39.0	−39.8 ± 6.4	*	−2.42 ± 0.04	−39.0	−31.9 ± 7.1	0.099
^{4}M2	−1.54 ± 0.02	−32.3			−0.74 ± 0.02	−23.3		
^{5}E	−2.52 ± 0.03	−48.0			−1.01 ± 0.03	−36.3		
^{5}E$_{58\text{-a/b}}$	−1.95 ± 0.01	−39.8			−0.55 ± 0.02	−28.8		
^{3}Vpu	−1.97 ± 0.07	−31.8	−30.8 ± 4.0	0.559	−1.97 ± 0.07	−31.8	−24.8 ± 7.3	0.531
^{3}M2	−1.26 ± 0.02	−26.4			−0.55 ± 0.02	−17.2		
^{3}E	−1.80 ± 0.03	−34.3			−0.71 ± 0.03	−25.4		
**ideal structures**	spp				lpp			
	Δ	Δ%	Ave. Δ%	*p*	Δ	Δ%	Ave. Δ%	*p*
^{3}S	−1.20 ± 0.03	−24.9	−28.9 ± 3.8	-	−1.20 ± 0.03	−24.9	−18.8 ± 5.3	-
^{3}HA	−1.23 ± 0.03	−28.3			−0.61 ± 0.02	−21.0		
^{3}gp41	−1.54 ± 0.04	−34.0			−0.45 ± 0.01	−23.5		
^{3}R696L	−1.47 ± 0.05	−31.7			−0.30 ± 0.02	−17.0		
^{3}gB	−1.24 ± 0.03	−25.5			−0.27 ± 0.01	−14.9		
^{5}Vpu	−3.04 ± 0.13	−49.4	−41.9 ± 6.3	*	−3.04 ± 0.13	−49.4	−33.3 ± 11	0.094
^{4}M2	−1.72 ± 0.02	−35.2			−0.95 ± 0.01	−27.9		
^{5}E	−2.22 ± 0.10	−44.3			−0.85 ± 0.03	−31.0		
^{5}Vpu$^{*}_{32}$	−1.74 ± 0.07	−38.6			−0.59 ± 0.02	−24.9		

Diffusion data derived from 500 ns MD simulations for the experimental and ideal conformations are calculated using the core TMD region of the TMDs (spp) and the whole number of amino acids (lpp). The translational values are given in [× 10^{-7} cm^2/s], the rotational diffusion data are as [1/μs]. The standard deviation is only given when calculated to be ≥ 0.01 units. ′p′ value is calculated pairwise using fusion protein as the reference. *: $p < 0.05$, **: $p < 0.01$.

Table S2: DCs derived from 500 ns MD simulations of the experimental and ideal single TMDs (monomer) and when simulated in their respective oligomeric states (oligomer).

Translational diffusion coefficient (experimental structures)						
**Monomer**	**spp**	**lpp**	**Δ (spp – lpp)**	**Δ%**	**aa length**	**Δ-length**
^{1}S	0.23	0.23	0.0	0.0 ± 0.3	21	0
^{1}HA	0.21	0.17 ± 0.01	−0.04	−18.3 ± 2.9	30	7
^{1}gB	0.22	0.14	−0.08	−34.7 ± 1.1	40	17
^{1}R696L	0.23	0.14 ± 0.01	−0.08	−36.9 ± 4.3	40	17
^{1}gp41	0.23	0.14	−0.09	−37.8 ± 2.5	40	17
^{1}Vpu	0.25	0.25	0.0	0.0 ± 0.3	19	0
^{1}M2	0.22	0.18	−0.04	−17.9 ± 0.6	28	5
^{1}E	0.23	0.17	−0.06	−27.2 ± 1.2	32	9
$^{1}E_{58\text{-}a/b}$	0.23	0.14	−0.09	−40.1 ± 1.4	58	25
Oligomer						
^{3}S	0.19	0.19	0.0	0.0 ± 2.3	21	0
^{3}HA	0.19	0.15	−0.04	−20.1 ± 1.4	30	7
^{3}gp41	0.19	0.13	−0.06	−30.1 ± 1.2	40	17
^{3}gB	0.19	0.13	−0.06	−32.6 ± 0.6	40	17
^{3}R696L	0.19	0.12	−0.07	−38.3 ± 0.5	40	17
^{5}Vpu	0.19	0.19	0.0	0.0 ± 1.0	19	0
^{4}M2	0.18	0.15	−0.03	−14.8 ± 0.7	28	5
^{5}E	0.17	0.13	−0.04	−23.5 ± 0.8	32	9
$^{5}E_{58\text{-}a/b}$	0.17	0.10	−0.07	−40.6 ± 1.4	58	25
^{3}Vpu	0.21	0.21	0.0	0.0 ± 1.6	19	0
^{3}M2	0.19	0.16	−0.03	−14.5 ± 0.4	28	5
^{3}E	0.19	0.14	−0.05	−25.0 ± 1.3	32	9

Translational diffusion coefficient (ideal structures)

Monomer	spp	lpp	Δ (spp – lpp)	Δ%	aa length	Δ-length
^{1}S	0.23	0.23	0.0	0.0 ± 0.6	21	0
^{1}HA	0.21	0.17	−0.04	−18.4 ± 0.9	30	7
^{1}gp41	0.22	0.14	−0.08	−37.0 ± 0.6	40	17
^{1}R696L	0.22	0.13	−0.09	−41.1 ± 1.7	40	17
^{1}gB	0.23	0.13	−0.09	−41.3 ± 0.7	40	17
^{1}Vpu	0.25	0.25	0.0	0.0 ± 1.7	19	0
^{1}M2	0.23	0.19	−0.04	−15.5 ± 0.4	28	5
^{1}E	0.23	0.17	−0.06	−25.4 ± 1.3	32	9
^{1}Vpu$^{*}_{32}$	0.22	0.16	−0.06	−28.2 ± 0.6	32	9
^{1}Vpu$^{*}_{53\text{-}a/b}$	0.21	0.12	−0.09	−43.2 ± 2.5	53	30
Oligomer						
^{3}S	0.20	0.20	0.0	0.0 ± 0.2	21	0
^{3}HA	0.18	0.15	−0.03	−16.1 ± 0.9	30	7
^{3}gp41	0.18	0.12	−0.06	−35.9 ± 0.4	40	17
^{3}R696L	0.18	0.11	−0.07	−37.9 ± 0.6	40	17
^{3}gB	0.19	0.12	−0.08	−40.2 ± 0.5	40	17
^{5}Vpu	0.19	0.19	0.0	0.0 ± 2.2	19	0
^{4}M2	0.18	0.15	−0.02	−13.2 ± 0.3	28	5
^{5}E	0.17	0.13	−0.04	−21.0 ± 0.4	32	9
^{5}Vpu$^{*}_{32}$	0.17	0.13	−0.04	−23.7 ± 1.1	32	9

Rotational diffusion coefficient (experimental structures)

Monomer	spp	lpp	Δ (spp – lpp)	Δ%	aa length	Δ-length
^{1}S	5.13 ± 0.04	5.13 ± 0.04	0.00 ± 0.02	0.0 ± 0.7	21	0
^{1}HA	4.17 ± 0.04	2.81 ± 0.15	−1.36 ± 0.07	−32.7 ± 5.3	30	7
^{1}gB	4.52 ± 0.04	2.04 ± 0.04	−2.48 ± 0.03	−54.9 ± 1.9	40	17

^{1}R696L	5.01 ± 0.20	2.09 ± 0.14	−2.92 ± 0.11	−58.3 ± 7.0	40	17
^{1}gp41	4.96 ± 0.18	2.01 ± 0.08	−2.95 ± 0.09	−59.5 ± 3.9	40	17
^{1}Vpu	6.20 ± 0.05	6.20 ± 0.05	0.00 ± 0.03	0.0 ± 0.8	19	0
^{1}M2	4.77 ± 0.03	3.17 ± 0.04	−1.60 ± 0.02	−33.5 ± 1.2	28	5
^{1}E	5.25 ± 0.01	2.78 ± 0.06	−2.47 ± 0.03	−47.0 ± 2.2	32	9
$^{1}E_{58\text{-}a/b}$	4.91 ± 0.03	1.90 ± 0.04	−3.01 ± 0.02	−61.3 ± 2.0	58	25
Oligomer						
^{3}S	3.29 ± 0.15	3.29 ± 0.15	0.00 ± 0.09	0.0 ± 4.7	21	0
^{3}HA	3.33 ± 0.09	2.23 ± 0.05	−1.10 ± 0.05	−32.9 ± 2.4	30	7
^{3}gp41	3.28 ± 0.03	1.79 ± 0.03	−1.49 ± 0.02	−45.4 ± 1.4	40	17
^{3}gB	3.56 ± 0.02	1.82 ± 0.02	−1.75 ± 0.01	−49.0 ± 0.9	40	17
^{3}R696L	3.47 ± 0.02	1.57 ± 0.01	−1.91 ± 0.01	−54.9 ± 0.7	40	17
^{5}Vpu	3.78 ± 0.08	3.78 ± 0.08	0.00 ± 0.05	0.0 ± 2.1	19	0
^{4}M2	3.23 ± 0.05	2.43 ± 0.03	−0.80 ± 0.02	−24.7 ± 1.3	28	5
^{5}E	2.73 ± 0.08	1.77 ± 0.02	−0.96 ± 0.04	−35.1 ± 1.4	32	9
$^{5}E_{58\text{-}a/b}$	2.96 ± 0.01	1.35 ± 0.02	−1.60 ± 0.01	−54.3 ± 1.2	58	25
^{3}Vpu	4.22 ± 0.14	4.22 ± 0.14	0.00 ± 0.09	0.0 ± 3.3	19	0
^{3}M2	3.51 ± 0.03	2.63 ± 0.02	−0.89 ± 0.01	−25.2 ± 0.6	28	5
^{3}E	3.45 ± 0.07	2.08 ± 0.04	−1.38 ± 0.04	−39.8 ± 2.0	32	9

Rotational diffusion (ideal structures)

**Monomer**	**spp**	**lpp**	**Δ (spp – lpp)**	**Δ%**	**aa length**	**Δ-length**
^{1}S	4.84 ± 0.06	4.84 ± 0.06	0.00 ± 0.04	0.0 ± 1.2	21	0
^{1}HA	4.33 ± 0.01	2.89 ± 0.04	−1.44 ± 0.02	−33.3 ± 1.5	30	7
^{1}gp41	4.53 ± 0.09	1.92 ± 0.02	−2.61 ± 0.04	−57.6 ± 0.9	40	17
^{1}R696L	4.64 ± 0.06	1.78 ± 0.04	−2.86 ± 0.03	−61.7 ± 2.4	40	17
^{1}gB	4.86 ± 0.05	1.82 ± 0.02	−3.04 ± 0.02	−62.5 ± 1.0	40	17

^{1}Vpu	6.14 ± 0.26	6.14 ± 0.26	0.00 ± 0.17	0.0 ± 4.4	19	0
^{1}M2	4.89 ± 0.04	3.42 ± 0.03	−1.47 ± 0.02	−30.0 ± 0.8	28	5
^{1}E	5.01 ± 0.22	2.75 ± 0.06	−2.25 ± 0.10	−45.0 ± 2.5	32	9
^{1}Vpu$^{*}_{32}$	4.51 ± 0.14	2.38 ± 0.02	−2.13 ± 0.06	−47.3 ± 1.0	32	9
^{1}Vpu$_{53\text{-}a/b}$	4.33 ± 0.07	1.62 ± 0.06	−2.71 ± 0.04	−62.6 ± 3.6	53	30
Oligomer						
^{3}S	3.63 ± 0.01	3.63 ± 0.01	0.00 ± 0.01	0.0 ± 0.3	21	0
^{3}HA	3.10 ± 0.07	2.28 ± 0.03	−0.82 ± 0.04	−26.4 ± 1.6	30	7
^{3}gp41	2.99 ± 0.02	1.47 ± 0.01	−1.52 ± 0.01	−50.9 ± 0.7	40	17
^{3}R696L	3.17 ± 0.09	1.47 ± 0.01	−1.69 ± 0.04	−53.5 ± 0.9	40	17
^{3}gB	3.62 ± 0.04	1.55 ± 0.01	−2.07 ± 0.02	−57.1 ± 0.6	40	17
^{5}Vpu	3.11 ± 0.13	3.11 ± 0.13	0.00 ± 0.08	0.0 ± 4.2	19	0
^{4}M2	3.16 ± 0.03	2.47 ± 0.01	−0.70 ± 0.01	−22.1 ± 0.6	28	5
^{5}E	2.79 ± 0.01	1.90 ± 0.01	−0.89 ± 0.01	−31.8 ± 0.6	32	9
^{5}Vpu$^{*}_{32}$	2.77 ± 0.06	1.79 ± 0.03	−0.98 ± 0.03	−35.5 ± 1.7	32	9

Calculations are done using the core TMD region of the TMDs (spp) and the whole number of amino acids (lpp). The entire length of amino acids used in the simulation is outlined (aa length) as well as the difference of the number of amino acids for the two type of calculations (Δ-length). The DC difference between spp and lpp is given in 'Δ'. 'Δ %' shows the changing percentage referring to the *spp* value as 100 %. The translational DCs are given in [$\times 10^{-7}$ cm^2/s], the rotational DCs are in [1/µs]. The standard deviation is only given when calculated to be ≥ 0.01 units.

Table S3. The *p*-values for the pairwise comparison of each of the DC calculated for the individual TMD either in the oligomeric (oligo) or monomeric (mono) form, using spp or lpp. The calculations are based on the values of the DCs as shown in Suppl. Table 2. The *p*-values are marked as blue boxes when < 0.05, yellow boxes when < 0.01 and red boxes when < 0.001. Grey boxes indicate that the values are ≥ 0.05. Duplicated values are omitted and represented as white boxes to avoid redundant information.

Translational diffusion (lpp, experimental structures)

**Mono**	**^{1}gp41**	**^{1}R696L**	**^{1}gB**	**^{1}S**	**^{1}HA**	**^{1}M2**	**^{1}E**	**$^{1}E_{58}$**	**^{1}Vpu**	**Mono**
^{1}gp41										^{1}gp41
^{1}R696L										^{1}R696L
^{1}gB										^{1}gB
^{1}S										^{1}S
^{1}HA										^{1}HA
^{1}M2										^{1}M2
^{1}E										^{1}E
$^{1}E_{58}$										$^{1}E_{58}$
^{1}Vpu										^{1}Vpu

Translational diffusion (lpp, ideal structures)

**Mono**	**^{1}gp41**	**^{1}R696L**	**^{1}gB**	**^{1}S**	**^{1}HA**	**^{1}M2**	**^{1}E**	**^{1}Vpu**	**$^{1}Vpu^{*}_{32}$**	**$^{1}Vpu^{*}_{53}$**	**Mono**
^{1}gp41											^{1}gp41
^{1}R696L											^{1}R696L
^{1}gB											^{1}gB
^{1}S											^{1}S
^{1}HA											^{1}HA
^{1}M2											^{1}M2
^{1}E											^{1}E
^{1}Vpu											^{1}Vpu
$^{1}Vpu^{*}_{32}$											$^{1}Vpu^{*}_{32}$
$^{1}Vpu^{*}_{53}$											$^{1}Vpu^{*}_{53}$

Translational diffusion (lpp, experimental structures)

**Oligo**	**^{3}gp41**	**^{3}R696L**	**^{3}gB**	**^{3}S**	**^{3}HA**	**^{4}M2**	**^{3}M2**	**^{5}E**	**^{3}E**	**$^{5}E_{58}$**	**^{5}Vpu**	**^{3}Vpu**	**Oligo**
^{3}gp41													^{3}gp41
^{3}R696L													^{3}R696L
gB													^{3}gB
^{3}S													^{3}S
^{3}HA													^{3}HA
^{4}M2													^{4}M2
^{3}M2													^{3}M2
^{5}E													^{5}E
^{3}E													^{3}E
$^{5}E_{58}$													$^{5}E_{58}$
^{5}Vpu													^{5}Vpu
^{3}Vpu													^{3}Vpu

Translational diffusion (lpp, ideal structures)

**Oligo**	**^{3}gp41**	**^{3}R696L**	**^{3}gB**	**^{3}S**	**^{3}HA**	**^{4}M2**	**^{5}E**	**^{5}Vpu**	**$^{5}Vpu^{*}_{32}$**	**Oligo**
^{3}gp41										^{3}gp41
^{3}R696L										^{3}R696L
^{3}gB										^{3}gB
^{3}S										^{3}S
^{3}HA										^{3}HA

^{4}M2										^{4}M2
^{5}E										^{5}E
^{5}Vpu										^{5}Vpu
^{5}Vpu$^{*}_{32}$										^{5}Vpu$^{*}_{32}$

Rotational diffusion (lpp, experimental structures)

**Mono**	**^{1}gp41**	**^{1}R696L**	**^{1}gB**	**^{1}S**	**^{1}HA**	**^{1}M2**	**^{1}E**	**^{1}E$_{58}$**	**^{1}Vpu**	**Mono**
^{1}gp41										^{1}gp41
^{1}R696L										^{1}R696L
^{1}gB										^{1}gB
^{1}S										^{1}S
^{1}HA										^{1}HA
^{1}M2										^{1}M2
^{1}E										^{1}E
^{1}E$_{58}$										^{1}E$_{58}$
^{1}Vpu										^{1}Vpu

Rotational diffusion (lpp, ideal structures)

**Mono**	**^{1}gp41**	**^{1}R696L**	**^{1}gB**	**^{1}S**	**^{1}HA**	**^{1}M2**	**^{1}E**	**^{1}Vpu**	**^{1}Vpu$^{*}_{32}$**	**^{1}Vpu$^{*}_{53}$**	**Mono**
^{1}gp41											^{1}gp41
^{1}R696L											^{1}R696L
^{1}gB											^{1}gB
^{1}S											^{1}S
^{1}HA											^{1}HA
^{1}M2											^{1}M2
^{1}E											^{1}E
^{1}Vpu											^{1}Vpu
^{1}Vpu$^{*}_{32}$											^{1}Vpu$^{*}_{32}$
^{1}Vpu$^{*}_{53}$											^{1}Vpu$^{*}_{53}$

Rotational diffusion (lpp, experimental structures)

**Oligo**	**^{3}gp41**	**^{3}R696L**	**^{3}gB**	**^{3}S**	**^{3}HA**	**^{4}M2**	**^{3}M2**	**^{5}E**	**^{3}E**	**^{5}E$_{58}$**	**^{5}Vpu**	**^{3}Vpu**	**Oligo**
^{3}gp41													^{3}gp41
^{3}R696L													^{3}R696L
^{3}gB													^{3}gB
^{3}S													^{3}S
^{3}HA													^{3}HA
^{4}M2													^{4}M2
^{3}M2													^{3}M2
^{5}E													^{5}E
^{3}E													^{3}E
^{5}E$_{58}$													^{5}E$_{58}$
^{5}Vpu													^{5}Vpu
^{3}Vpu													^{3}Vpu

Rotational diffusion (lpp, ideal structures)

**Oligo**	**^{3}gp41**	**^{3}R696L**	**^{3}gB**	**^{3}S**	**^{3}HA**	**^{4}M2**	**^{5}E**	**^{5}Vpu**	**^{5}Vpu$^{*}_{32}$**	**Oligo**
^{3}gp41										^{3}gp41
^{3}R20L										^{3}R696L
^{3}gB										^{3}gB
^{3}S										^{3}S
^{3}HA										^{3}HA
^{4}M2										^{4}M2
^{5}E										^{5}E
^{5}Vpu										^{5}Vpu
^{5}Vpu$^{*}_{32}$										^{5}Vpu$^{*}_{32}$

Translational diffusion (spp, experimental structures)

Mono	^{1}gp41	^{1}R696L	^{1}gB	^{1}S	^{1}HA	^{1}M2	^{1}E	^{1}E$_{58}$	^{1}Vpu	Mono
^{1}gp41										^{1}gp41
^{1}R696L										^{1}R696L
^{1}gB										^{1}gB
^{1}S										^{1}S
^{1}HA										^{1}HA
^{1}M2										^{1}M2
^{1}E										^{1}E
^{1}E$_{58}$										^{1}E$_{58}$
^{1}Vpu										^{1}Vpu

Translational diffusion (spp, ideal structures)

Mono	^{1}gp41	^{1}R696L	^{1}gB	^{1}S	^{1}HA	^{1}M2	^{1}E	^{1}Vpu	^{1}Vpu$^{*}_{32}$	^{1}Vpu$^{*}_{53}$	Mono
^{1}gp41											^{1}gp41
^{1}R696L											^{1}R696L
^{1}gB											^{1}gB
^{1}S											^{1}S
^{1}HA											^{1}HA
^{1}M2											^{1}M2
^{1}E											^{1}E
^{1}Vpu											^{1}Vpu
^{1}Vpu$^{*}_{32}$											^{1}Vpu$^{*}_{32}$
^{1}Vpu$^{*}_{53}$											^{1}Vpu$^{*}_{53}$

Translational diffusion (spp, experimental structures)

Oligo	^{3}gp41	^{3}R696L	^{3}gB	^{3}S	^{3}HA	^{4}M2	^{3}M2	^{5}E	^{3}E	^{5}E$_{58}$	^{5}Vpu	^{3}Vpu	Oligo
^{3}gp41													^{3}gp41
^{3}R696L													^{3}R696L
gB													^{3}gB
^{3}S													^{3}S
^{3}HA													^{3}HA
^{4}M2													^{4}M2
^{3}M2													^{3}M2
^{5}E													^{5}E
^{3}E													^{3}E
^{5}E$_{58}$													^{5}E$_{58}$
^{5}Vpu													^{5}Vpu
^{3}Vpu													^{3}Vpu

Translational diffusion (spp, ideal structures)

Oligo	^{3}gp41	^{3}R696L	^{3}gB	^{3}S	^{3}HA	^{4}M2	^{5}E	^{5}Vpu	^{5}Vpu$^{*}_{32}$	Oligo
^{3}gp41										^{3}gp41
^{3}R696L										^{3}R696L
^{3}gB										^{3}gB
^{3}S										^{3}S
^{3}HA										^{3}HA
^{4}M2										^{4}M2
^{5}E										^{5}E
^{5}Vpu										^{5}Vpu
^{5}Vpu$^{*}_{32}$										^{5}Vpu$^{*}_{32}$

Rotational diffusion (spp, experimental structures)

Mono	^{1}gp41	^{1}R696L	^{1}gB	^{1}S	^{1}HA	^{1}M2	^{1}E	^{1}E$_{58}$	^{1}Vpu	Mono
^{1}gp41										^{1}gp41
^{1}R696L										^{1}R696L

^{1}gB										^{1}gB
^{1}S										^{1}S
^{1}HA										^{1}HA
^{1}M2										^{1}M2
^{1}E										^{1}E
^{1}E$_{58}$										^{1}E$_{58}$
^{1}Vpu										^{1}Vpu

Rotational diffusion (spp, ideal structures)

**Mono**	**^{1}gp41**	**^{1}R696L**	**^{1}gB**	**^{1}S**	**^{1}HA**	**^{1}M2**	**^{1}E**	**^{1}Vpu**	**^{1}Vpu$^{*}_{32}$**	**^{1}Vpu$^{*}_{53}$**	**Mono**
^{1}gp41											^{1}gp41
^{1}R696L											^{1}R696L
^{1}gB											^{1}gB
^{1}S											^{1}S
^{1}HA											^{1}HA
^{1}M2											^{1}M2
^{1}E											^{1}E
^{1}Vpu											^{1}Vpu
^{1}Vpu$^{*}_{32}$											^{1}Vpu$^{*}_{32}$
^{1}Vpu$^{*}_{53}$											^{1}Vpu$^{*}_{53}$

Rotational diffusion (spp, experimental structures)

**Oligo**	**^{3}gp41**	**^{3}R696L**	**^{3}gB**	**^{3}S**	**^{3}HA**	**^{4}M2**	**^{3}M2**	**^{5}E**	**^{3}E**	**^{5}E$_{58}$**	**^{5}Vpu**	**^{3}Vpu**	**Oligo**
^{3}gp41													^{3}gp41
^{3}R696L													^{3}R696L
^{3}gB													^{3}gB
^{3}S													^{3}S
^{3}HA													^{3}HA
^{4}M2													^{4}M2
^{3}M2													^{3}M2
^{5}E													^{5}E
^{3}E													^{3}E
^{5}E$_{58}$													^{5}E$_{58}$
^{5}Vpu													^{5}Vpu
^{3}Vpu													^{3}Vpu

Rotational diffusion (spp, ideal structures)

**Oligo**	**^{3}gp41**	**^{3}R696L**	**^{3}gB**	**^{3}S**	**^{3}HA**	**^{4}M2**	**^{5}E**	**^{5}Vpu**	**^{5}Vpu$^{*}_{32}$**	**Oligo**
^{3}gp41										^{3}gp41
^{3}R20L										^{3}R696L
^{3}gB										^{3}gB
^{3}S										^{3}S
^{3}HA										^{3}HA
^{4}M2										^{4}M2
^{5}E										^{5}E
^{5}Vpu										^{5}Vpu
^{5}Vpu$^{*}_{32}$										^{5}Vpu$^{*}_{32}$

Table S4. Radii of fitted curves to the standard deviation of the rotational dynamics for a selected sequence of amino acids from experimental (exp) and ideal peptides (ideal).

Exp	Mono	Oligomer							Difference (%)
**peptides**		**A**	**B**	**C**	**D**	**E**	**Ave.**	**Note**	**Ave.**
^{3}S	8.5	13.4	12.2	14.0			13.2 ± 0.9		54.5 ± 11
^{3}gp41	7.6	9.5	13.5	14.9			12.7 ± 2.8		65.8 ± 37
^{3}R696L	13.4	9.6	14.5	9.6			11.2 ± 2.8		−15.9 ± 21
^{3}gB	18.1	24.7	17.1	15.1			19.0 ± 5.1		5.1 ± 28
^{3}HA	11.3	15.0	9.7	18.5			14.4 ± 4.4		27.3 ± 39
^{4}M2	12.8							0/3	↑
^{5}E	15.4	25.6				19.9		2/5	↑
$^{5}E_{58\text{-}a/b}$	13.4							0/5	↑
	18.2	12.9		8.8				2/5	↑
^{5}Vpu	11.8							0/5	↑
^{3}M2	12.8	15.9						1/3	↑
^{3}E	15.4	15.8	28.2					2/3	↑
^{3}Vpu	11.8		14.4					1/3	↑
**Ideal**	**Mono**	**Oligomer**							**Difference (%)**
**peptides**		**A**	**B**	**C**	**D**	**E**	**Ave.**	**Note**	**Ave.**
^{3}S	9.7	13.0	11.0	17.2			13.7 ± 3.1		42.2 ± 33
^{3}gp41	10.1	12.5	19.7	9.9			14.0 ± 5.0		38.5 ± 50
^{3}R696L	14.4	15.7	9.4	19.7			14.9 ± 5.2		3.5 ± 36
^{3}gB	26.3	14.5	13.6	15.7			14.6 ± 1.0		−44.6 ± 4
^{3}HA	11.7	14.1	14.3	7.4			12.0 ± 3.9		2.7 ± 34
^{4}M2	11.5							0/4	↑
^{5}E	10.1			12.8		15.7		2/5	↑
^{5}Vpu	12.1		22.8		43.0			2/5	↑
$^{5}Vpu^{*}_{32}$	11.7							0/5	↑
**Ideal**	**Mono**	**Oligomer**							**Difference (%)**
**peptides**		**A**	**B**	**C**	**D**	**E**	**Ave.**	**Note**	**Ave.**
$^{1}Vpu^{*}_{53a/b}$	18.2								
	16.0								
$^{3}Vpu^{*}_{32}$	11.7		18.6	28.4				2/3	↑
$^{4}Vpu^{*}_{32}$	11.7	19.9	30.1	26.6	16.9		23.4 ± 6.0	4/4	99.8 ± 51
$^{5}Vpu^{*}_{32}$	11.7							0/5	↑
$^{5}Vpu^{*}_{32\text{-}r26}$	11.7							0/5	↑

The sequence of the individual TMDs are marked as red boxes in Figure 5, and Supplementary Figures S5A-F. Fittings are done on the monomers (mono) and oligomers. A-E indicate the individual helices of the oligomers. The oligomer radii are averaged (ave.) and the difference (ave. − mono) is expressed as a percentage with reference to the mono-values. For the yellow boxes, the values are $> 5 \times 10^4$ units (up to 9.4×10^5 units). The upward pointing arrow indicates values that are $> 7 \times 10^5$ % (up to 4.4×10^6 %). 'Note' indicates the number of TMDs out of the total number of TMDs of the oligomer, which have small radii, e.g. 2 out of 5 (2/5). If the difference is larger than 0, the curvature is lowered, if smaller than 0, the curvature is increased.

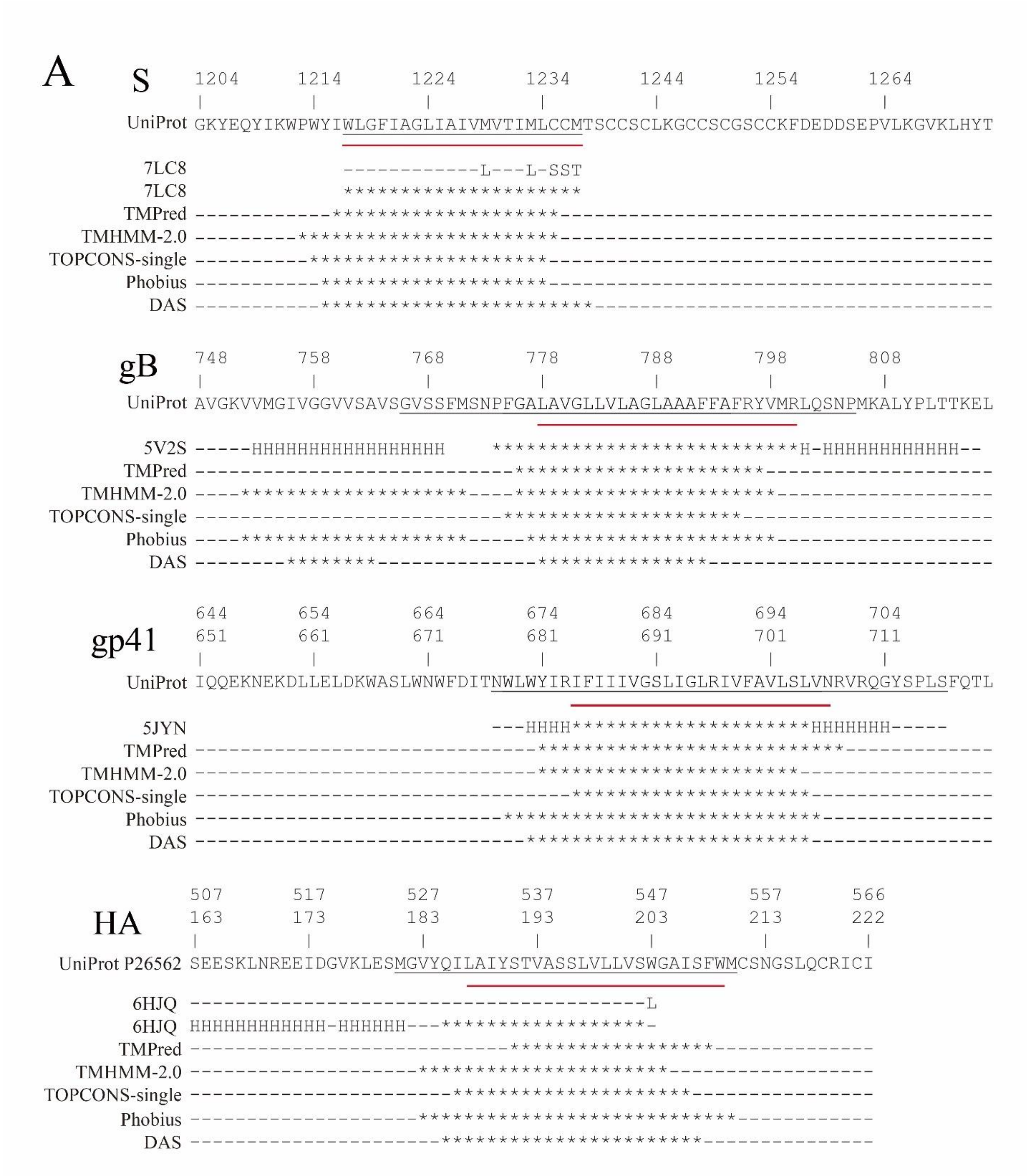
A
S
1204 1214 1224 1234 1244 1254 1264
UniProt GKYEQYIKWPWYIWLGFIAGLIAIVMVTIMLCCMTSCCSCLKGCCSCGSCCKFDEDDSEPVLKGVKLHYT
7LC8
7LC8
TMPred
TMHMM-2.0
TOPCONS-single
Phobius
DAS
gB
748 758 768 778 788 798 808
UniProt AVGKVVMGIVGGVVSAVSGVSSFMSNPFGALAVGLLVLAGLAAAFFAFRYVMRLQSNPMKALYPLTTKEL
5V2S
TMPred
TMHMM-2.0
TOPCONS-single
Phobius
DAS
gp41
644 654 664 674 684 694 704
651 661 671 681 691 701 711
UniProt IQQEKNEKDLLELDKWASLWNWFDITNWLWYIRIFIIIVGSLIGLRIVFAVLSLVNRVRQGYSPLSFQTL
5JYN
TMPred
TMHMM-2.0
TOPCONS-single
Phobius
DAS
HA
507 517 527 537 547 557 566
163 173 183 193 203 213 222
UniProt P26562 SEESKLNREEIDGVKLESMGVYQILAIYSTVASSLVLLVSWGAISFWMCSNGSLQCRICI
6HJQ
6HJQ
TMPred
TMHMM-2.0
TOPCONS-single
Phobius
DAS

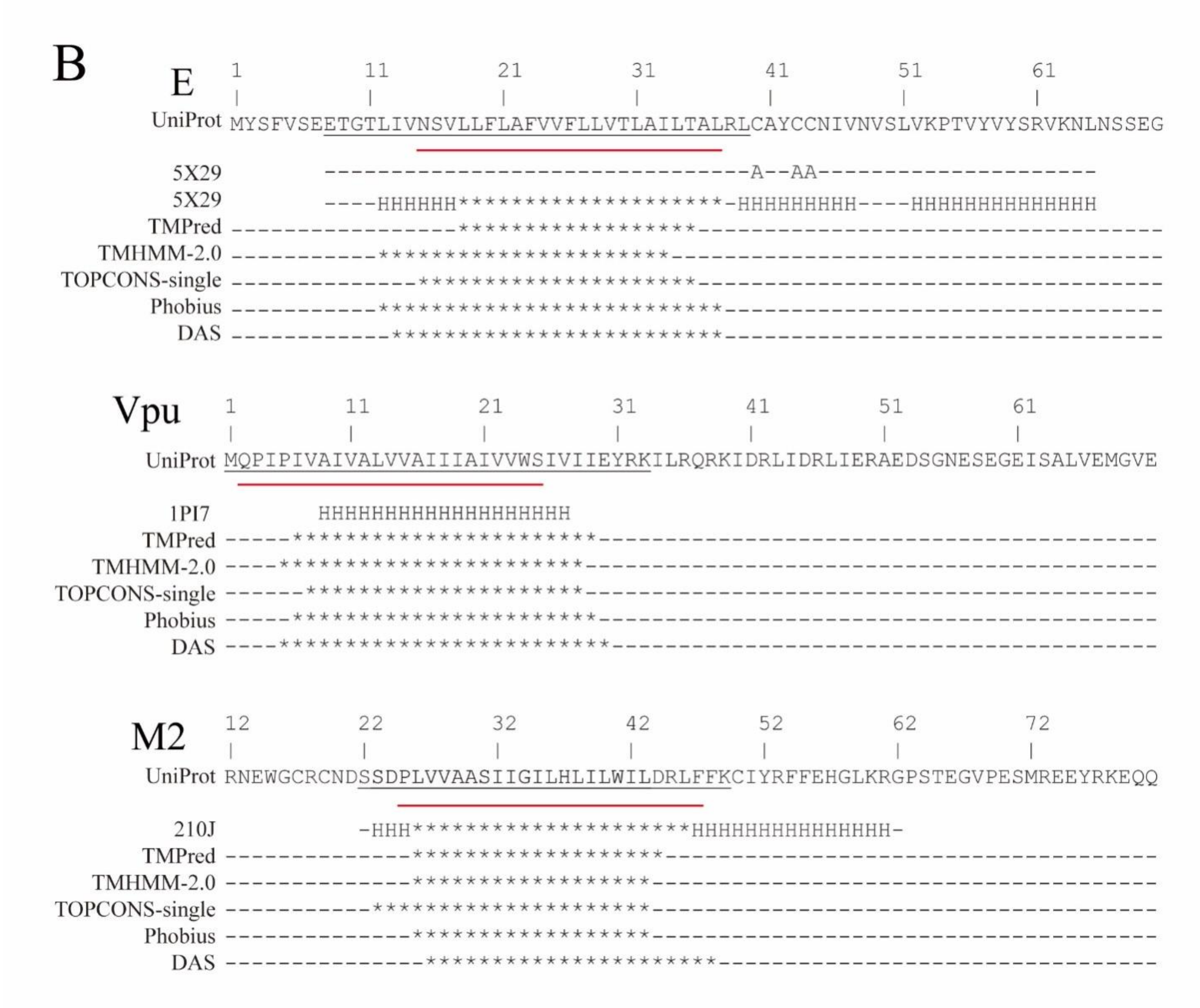

Figure S1. Prediction of helical motifs in the transmembrane domains of (**A**) the fusion peptides (S, gB, HA, gp41) and (**B**) ion channel peptides (E, Vpu, M2). The sequences are taken from UniProt. Altered sequences used in the experiments are outlined and marked by the PDB ID code (also for E_{58}, which is from 5X29). Residues proposed by the secondary structure prediction programs TMPred, TMHMM-2.0, TOPCONS-single, Phobius and DAS are shown by stars. Stars labeled after the PDB ID indicate the transmembrane domain, which is identified experimentally. H indicates a helical motif experimentally identified outside the membrane. Underlined residues are used in the MD simulations. The red line marks those residues, which are used for the calculation of the diffusion coefficients according to spp.

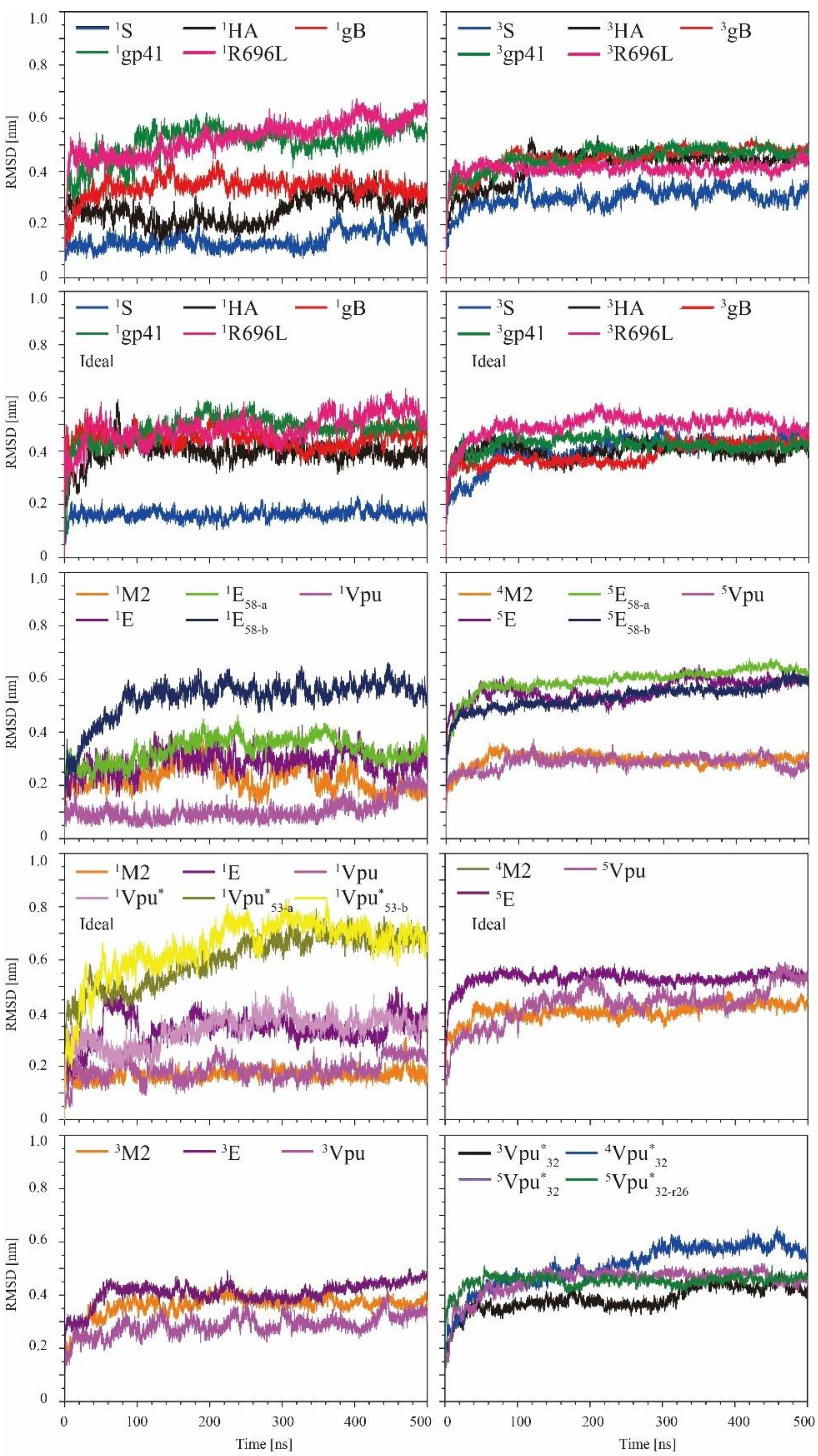

Figure S2. Root mean square deviation (RMSD) calculated for each of the peptides used in a 500 ns MD simulation. Superscripts in front of the letters mark the oligomeric state. Subscripts mark the specific length of the peptides. For Vpu$^{*}_{32}$: r marks the rank (r) of the structures when using PICA to generate the pentameric bundle, e.g. r26. For ^{5}Vpu$^{*}_{32}$, the tryptophan residues are pointing inside the pore, and 'r26' marks the pentameric bundle with the tryptophan residues are pointing outside the bundle leaving the S24 inside the putative pore, as well as in the trimeric and tetrameric bundles. 'Ideal' indicates that the peptides without stars are ideal structures derived from the respective experimental structures.

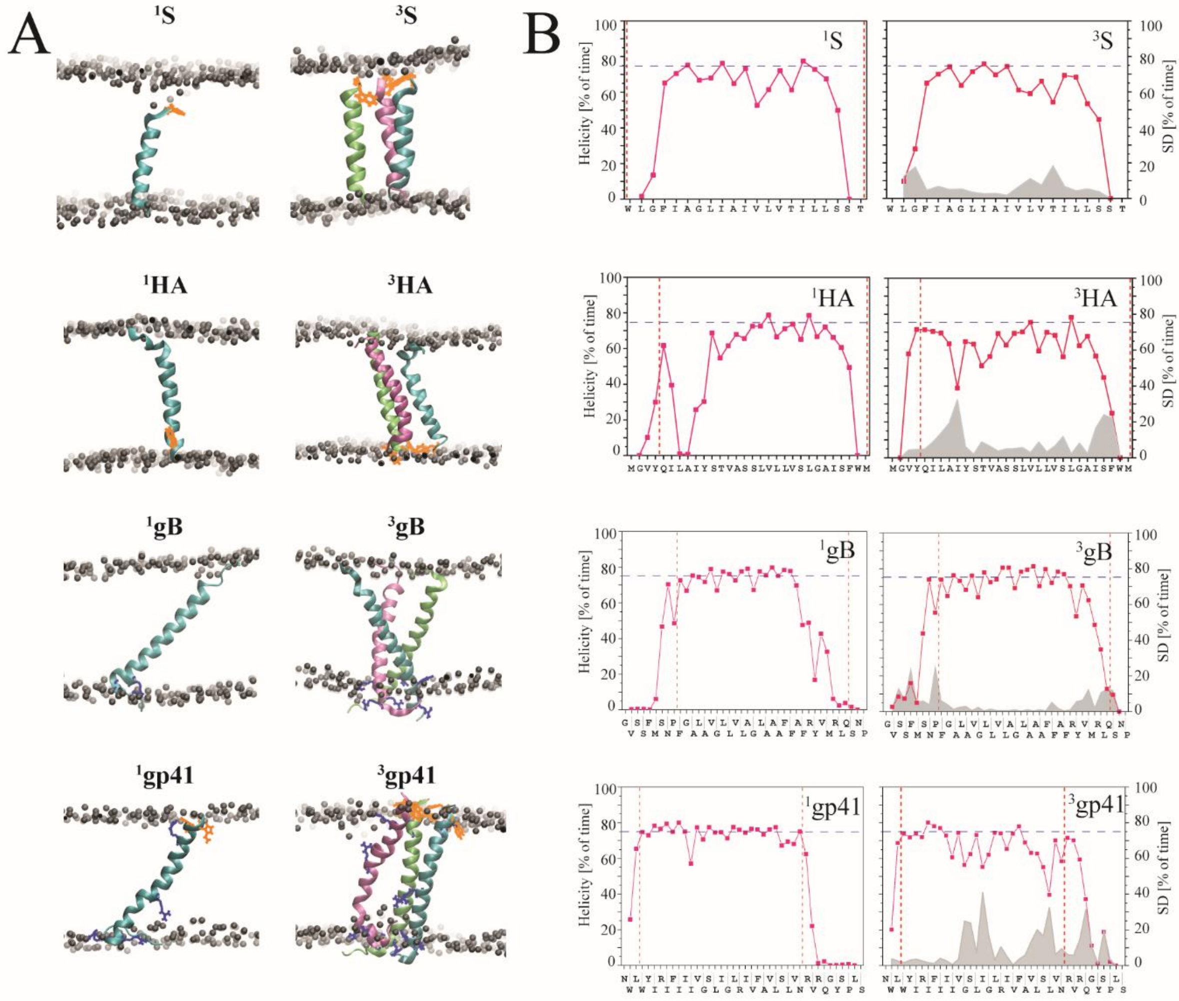
A
1S
3S
1HA
3HA
1gB
3gB
1gp41
3gp41
B
Helicity [% of time]
SD [% of time]
W L G F I A G L I A I V L V T I L L S S T
M G V Y Q I L A I Y S T V A S S L V L L V S L G A I S F W M
G S F S P G L V L V A L A F A R V R Q N
V S M N F A A G L L G A A F F Y M L S P
N L Y R F I V S I L I F V S V R R G S L
W W I I I I G L G R V A L L N V Q Y P S

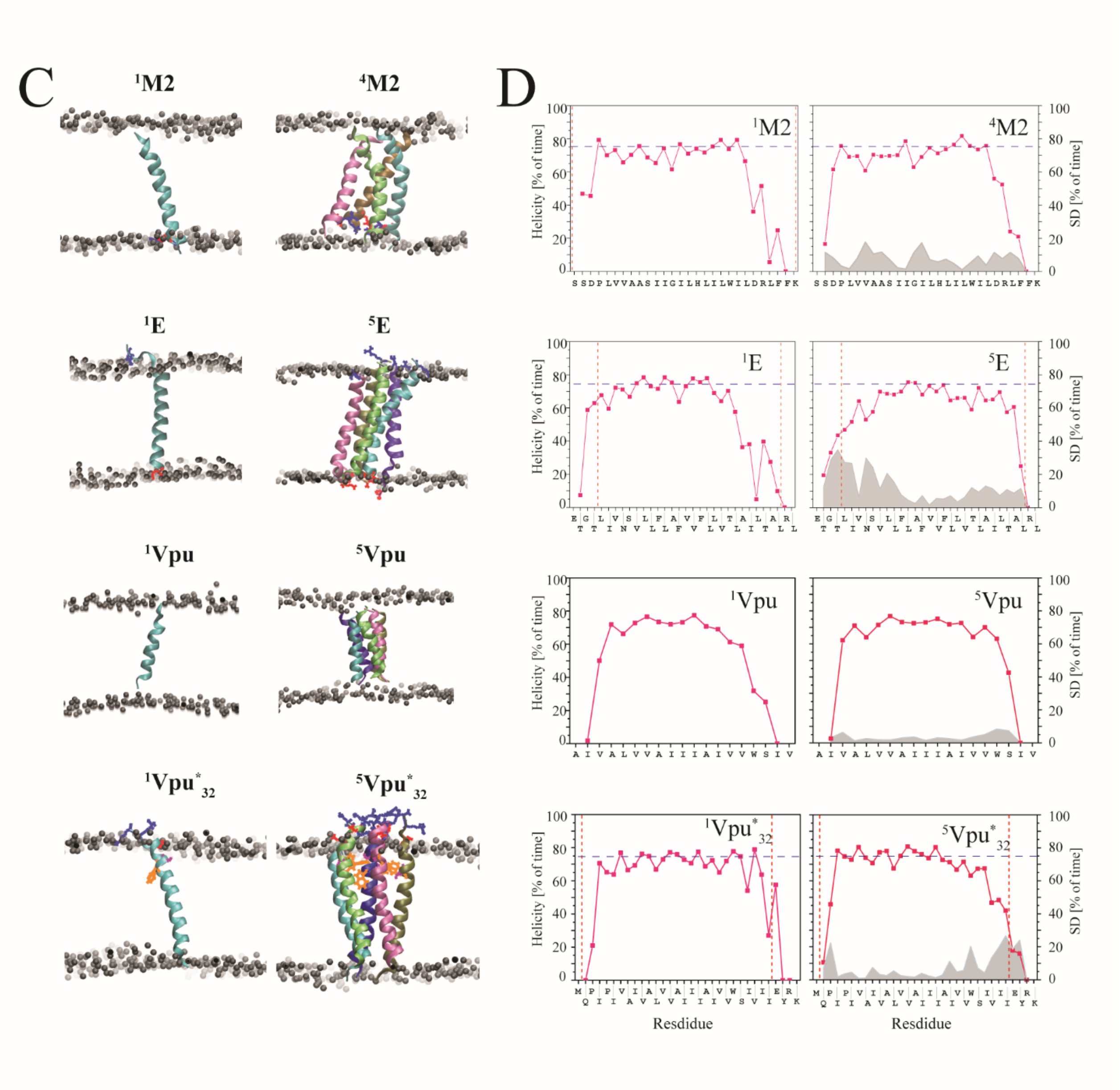

C
^{1}M2
^{4}M2
^{1}E
^{5}E
^{1}Vpu
^{5}Vpu
^{1}Vpu$^*_{32}$
^{5}Vpu$^*_{32}$
D
Helicity [% of time]
SD [% of time]
S S D P L V V A A S I I G I L H L I L W I L D R L F F K
E G L V S L F A V F L T A L A R
T T I N V L L F V L V L I T L L
A I V A L V V A I I I A I V V W S I V
M P P V I A V A I A V W I I E R
Q I I A V L V I I I V S V I Y K
Resdidue

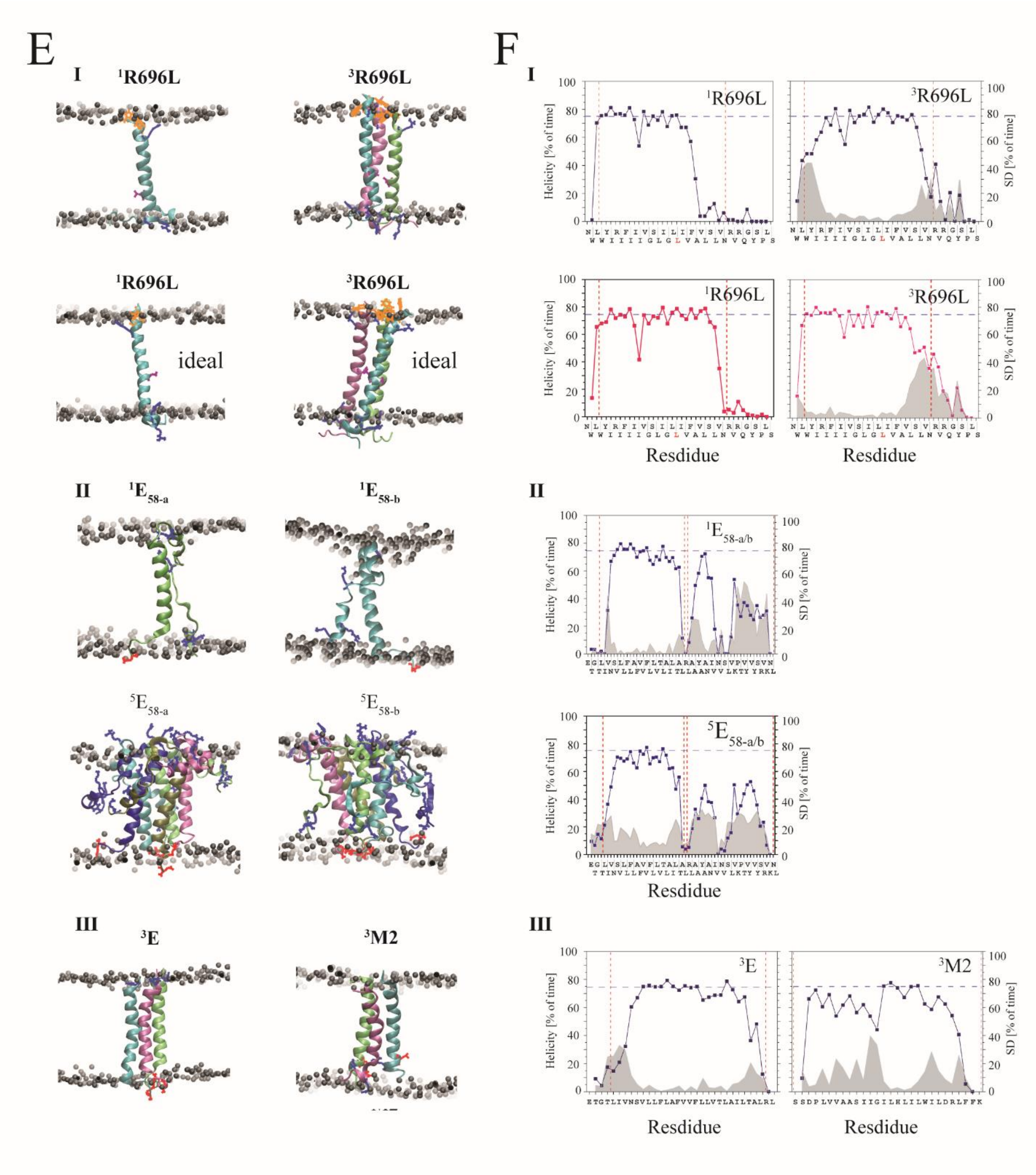
E
F
I
¹R696L
³R696L
¹R696L
³R696L
ideal
ideal
II
¹E58-a
¹E58-b
⁵E58-a
⁵E58-b
¹E58-a/b
⁵E58-a/b
III
³E
³M2
Helicity [% of time]
SD [% of time]
Resdidue

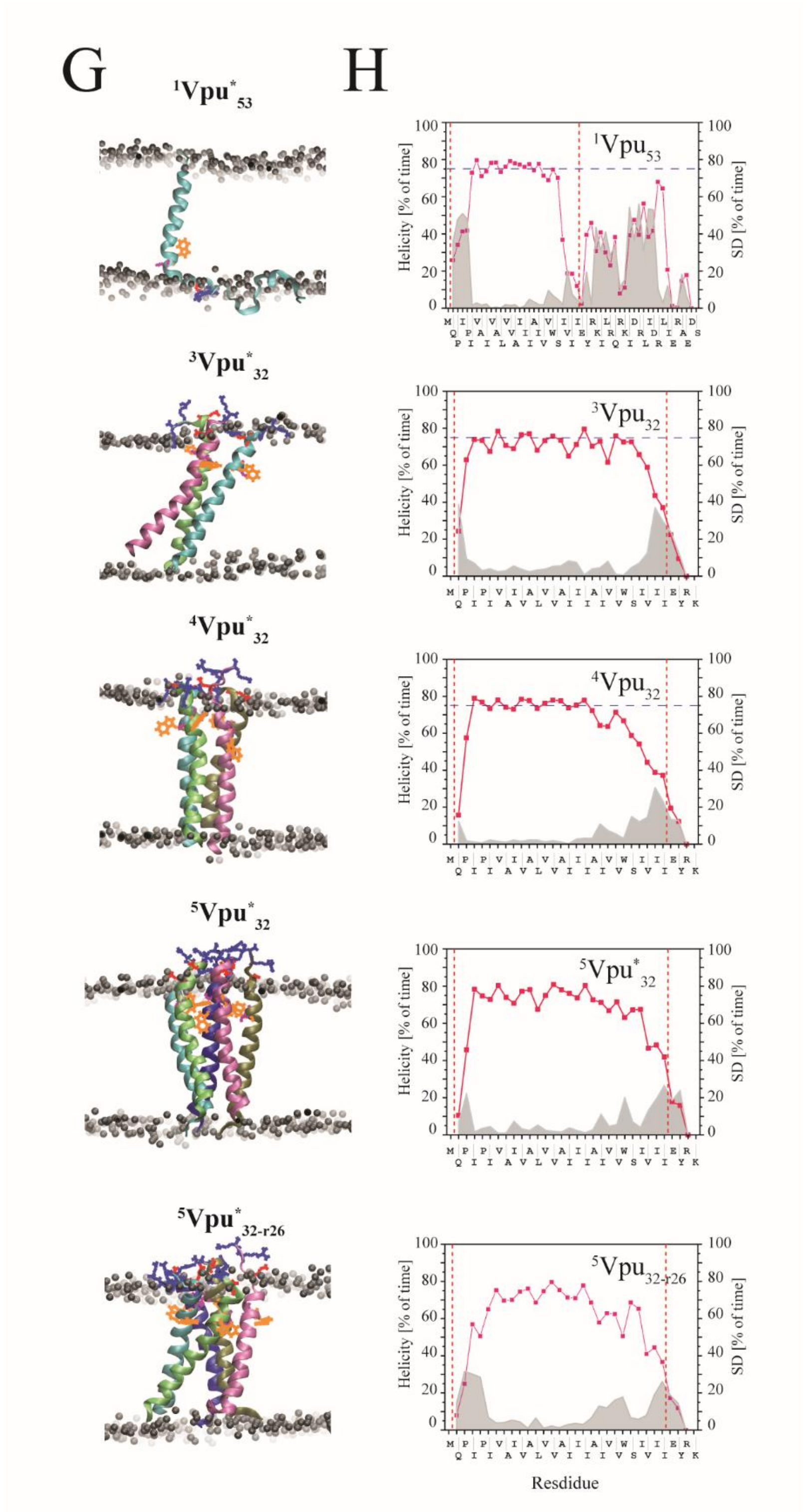

Figure S3. (**A**) Ideal structural models of the peptides in the monomeric and oligomeric state at the end of the 500 ns MD simulation of S (^{1}S, ^{3}S), HA (^{1}HA, ^{3}HA), gB (^{1}gB, ^{3}gB), and gp41 (^{1}gp41, ^{3}gp41). The boundaries of the lipid membrane are marked by grey spheres representing the phosphorous atoms of the headgroups. The remaining lipid atoms as well as the water molecules are omitted for clarity. (**B**) The corresponding percentage of time of each residue being in a helical conformation is shown in the plots for both monomer (left column) and oligomer with standard deviation in grey when averaging over the values for each individual TMD (right column). Red dash lines indicate the approximate boundaries of the lipid membrane. (**C**) Ideal structural models of the peptides in

the monomeric and oligomeric state at the end of the 500 ns MD simulation of M2 (^{1}M2, ^{4}M2), E peptide (^{1}E, ^{5}E) and Vpu peptide (^{1}Vpu, ^{5}Vpu, ^{1}Vpu$^{*}_{32}$, ^{5}Vpu$^{*}_{32}$); (**D**) as in (B). (**E**, **F**) I: Experimental structural data and helicity data (blue lines) for $^{1/3}$R696L and ideal $^{1/3}$R696L (red lines in the helicity plots). II: Experimental structural models of the peptides in the monomeric and oligomeric state at the end of the 500 ns MD simulation of $^{1/5}$E$_{58\text{-}a}$ and $^{1/5}$E$_{58\text{-}b}$. The helicity plot shows the average values between $^{1/5}$E$_{58\text{-}a}$ and $^{1/5}$E$_{58\text{-}b}$. III: Artificial trimeric structural models and helicity data for ^{3}E and ^{3}M2; the arrangement and color coding of (**F**) are as described in (**B**). (**G**) Ideal structural models of the peptides in the monomeric and oligomeric state at the end of the 500 ns MD simulation of Vpu peptides: ^{1}Vpu$^{*}_{53}$, ^{3}Vpu$^{*}_{32}$, ^{4}Vpu$^{*}_{32}$, ^{5}Vpu$^{*}_{32}$ and ^{5}Vpu$^{*}_{32\text{-}r26}$. (**H**) The corresponding percentage of time of each residue being in a helical conformation with standard deviation when averaging over the values for each individual TMD in the oligomer. Red dash lines indicate the lipid membrane boundary. 'r26' marks the rank (r) of the structures when using PICA to generate the pentameric bundle. For ^{5}Vpu$^{*}_{32}$ the tryptophan residues are pointing inside the pore, and 'r26' marks the pentameric bundle with the tryptophan residues are pointing outside the bundle leaving the S24 inside the putative pore, as well as in the trimeric and tetrameric bundles.

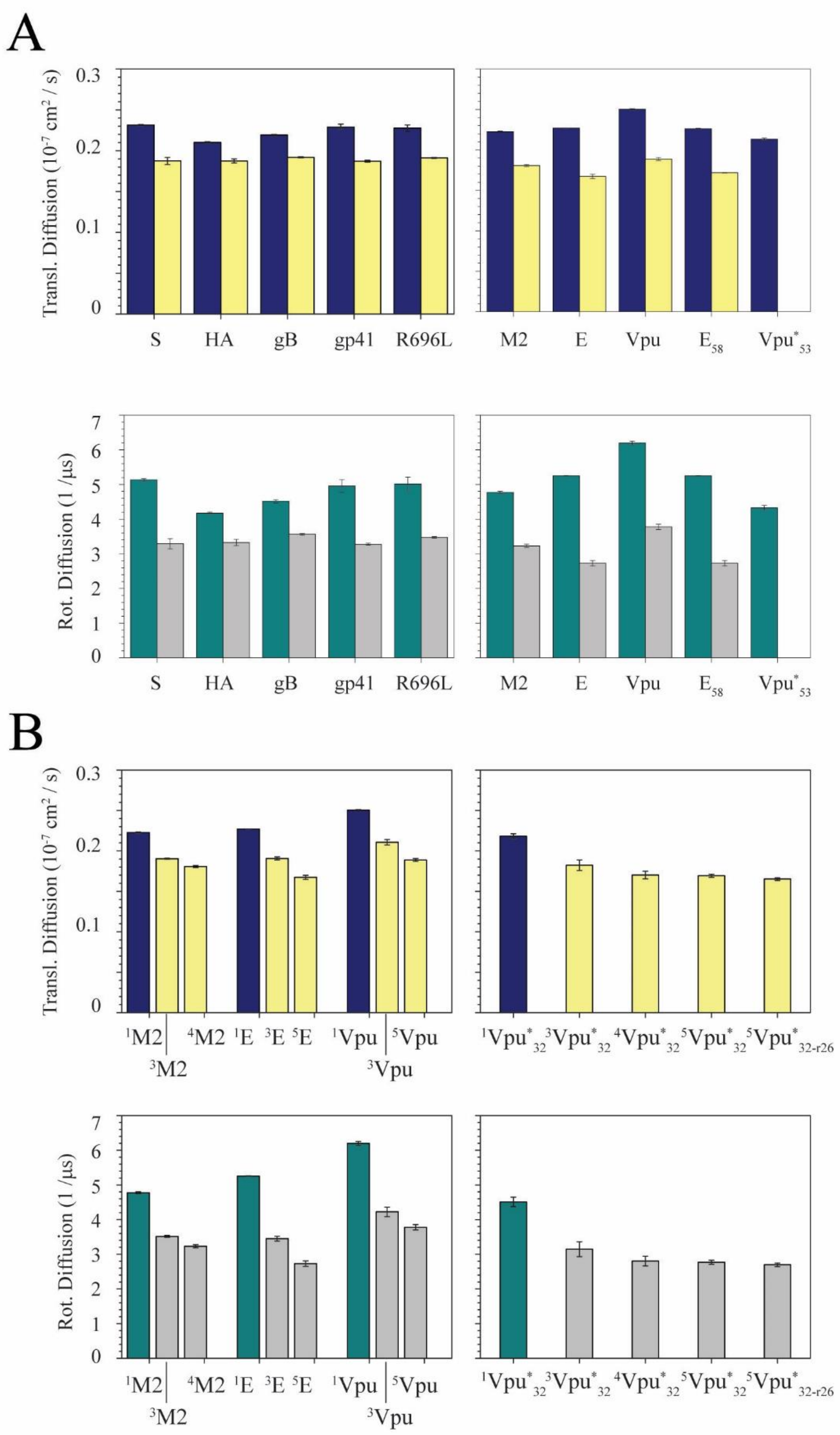
A
Transl. Diffusion (10-7 cm2 / s)
0.3
0.2
0.1
0
S
HA
gB
gp41
R696L
M2
E
Vpu
E58
Vpu*53
Rot. Diffusion (1 /µs)
7
6
5
4
3
2
1
0
B
1M2
3M2
4M2
1E
3E
5E
1Vpu
3Vpu
5Vpu
1Vpu*32
3Vpu*32
4Vpu*32
5Vpu*32
5Vpu*32-r26

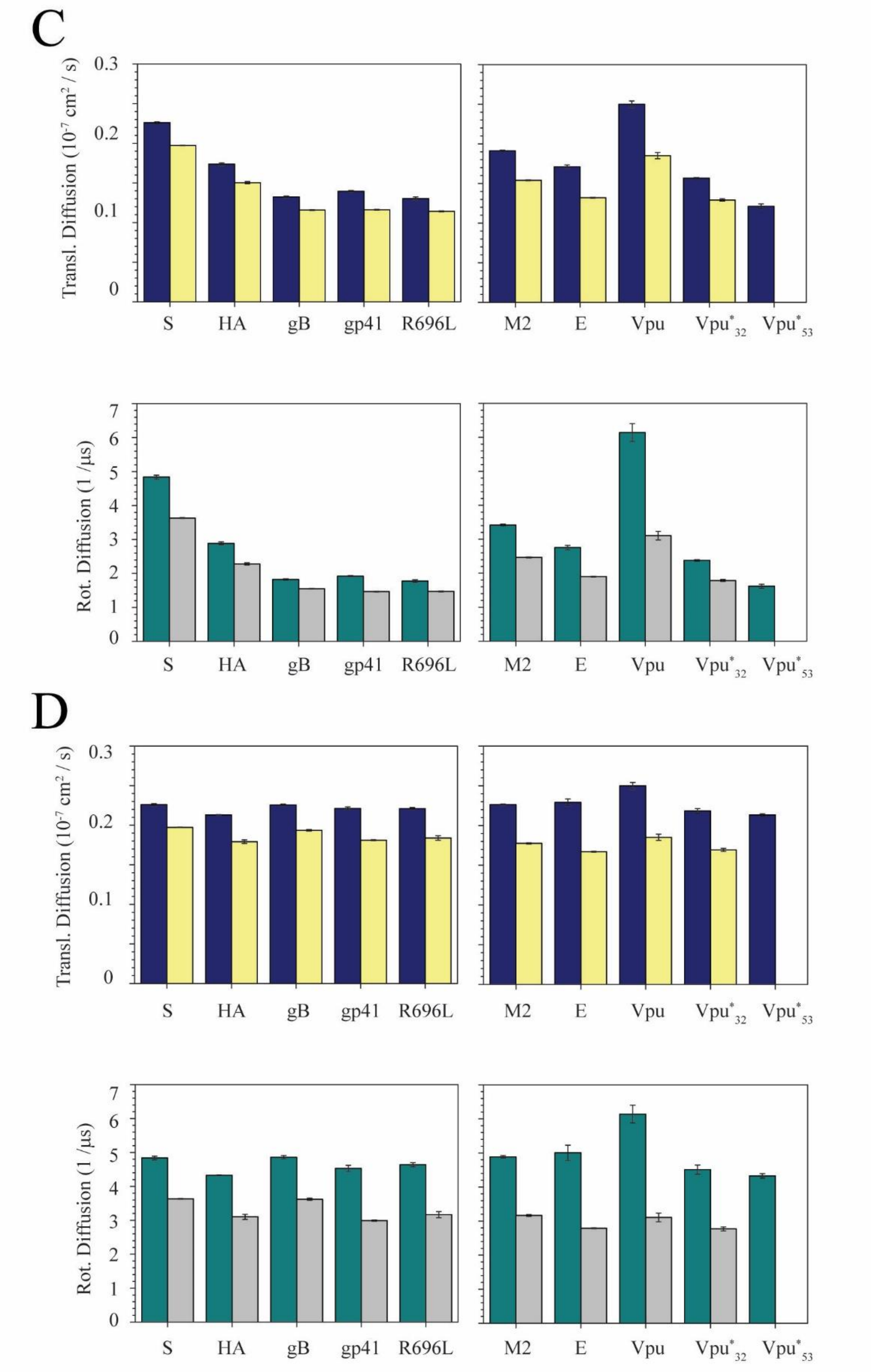

Figure S4. (**A**) Translational (upper panel) and rotational diffusion (lower panel) of fusion (left column) and channel peptides (right column) from MD simulations of the experimental structures using spp. The blue and green bars represent the data for monomers, while the yellow and grey bars represent the data of the oligomers (3 TMDs for each fusion protein; 4 TMDs for M2, and 5 TMDs for E and Vpu proteins). The star in [1]Vpu*$_{53}$ indicates that this structure is derived from an

ideal helix. The subscripts for Vpu and E protein mark the number of amino acids used in the simulation. (**B**) Diffusion data for spp from specific oligomeric structures generated by PICA. The color scheme is the same as above. Left column shows the diffusion coefficients of the experimentally derived ion channels in monomeric and tetra/pentameric state together with PICA generated trimeric conformations by using experimental monomers as building blocks. Right column shows the PICA generated oligomeric bundles by using ^{1}Vpu$^{*}_{32}$. The superscripts mark the oligomeric state, which was used in the simulations. 'r26' marks the rank (r) of the structures when PICA is used to generate the oligomeric state. For ^{5}Vpu$^{*}_{32}$, the tryptophan residues are pointing inside the pore, while for ^{5}Vpu$^{*}_{32\text{-}r26}$ the tryptophan residues are pointing outside the bundle, leaving the S24 pointing inside the putative pore, as well as in the trimeric and tetrameric bundles. (**C**) Translational (upper panel) and rotational diffusion coefficients (DCs) (lower panel) of fusion (left column) and channel peptides (right column) from MD simulations of the ideal structures. The data are calculated using lpp. The blue and green bars represent the data for the monomers, while the yellow and grey bars represent the data of the oligomers. The subscripts for Vpu and E protein mark the number of amino acids used in the simulation. (**D**) DCs for structures using spp. The same color scheme as above is applied. The superscripts mark the oligomeric state, which was used in the simulations.

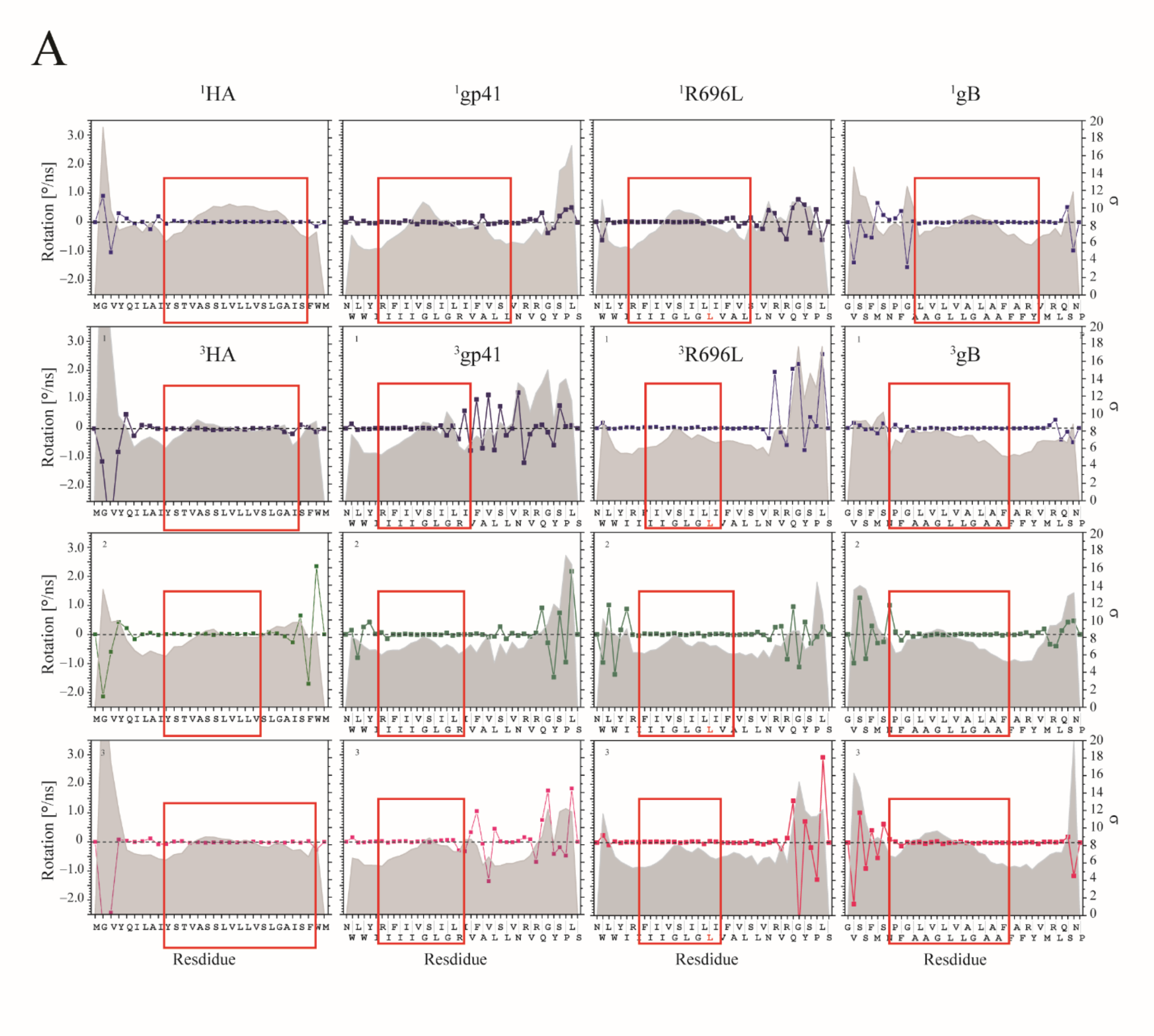

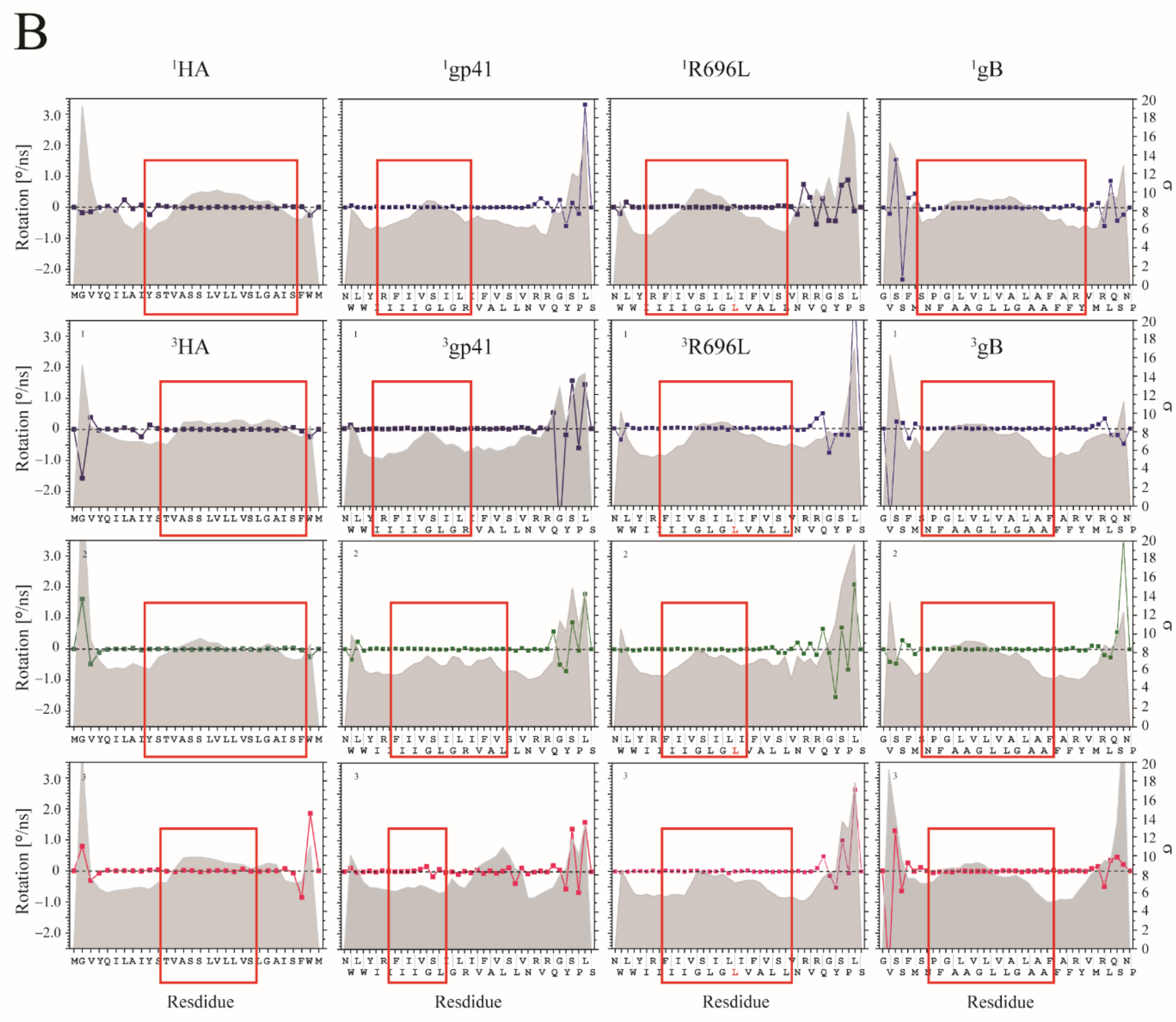
B
¹HA
¹gp41
¹R696L
¹gB
³HA
³gp41
³R696L
³gB
Rotation [°/ns]
3.0
2.0
1.0
0
−1.0
−2.0
σ
20
18
16
14
12
10
8
6
4
2
0
Resdidue

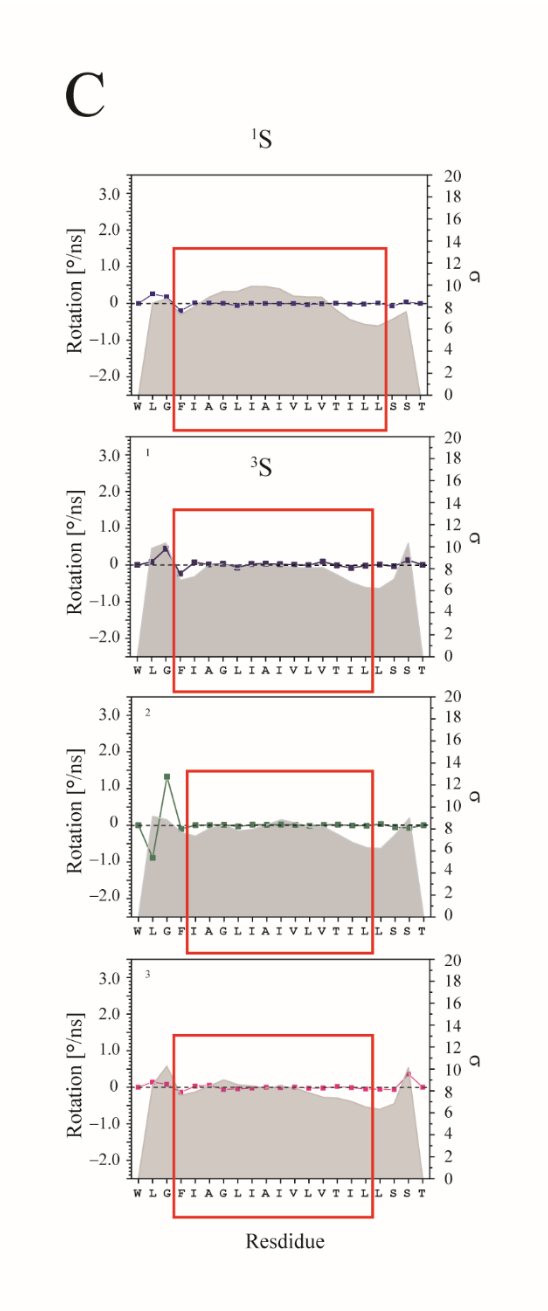
C
¹S
Rotation [°/ns]
σ
W L G F I A G L I A I V L V T I L L S S T
³S
1
2
3
Resdidue

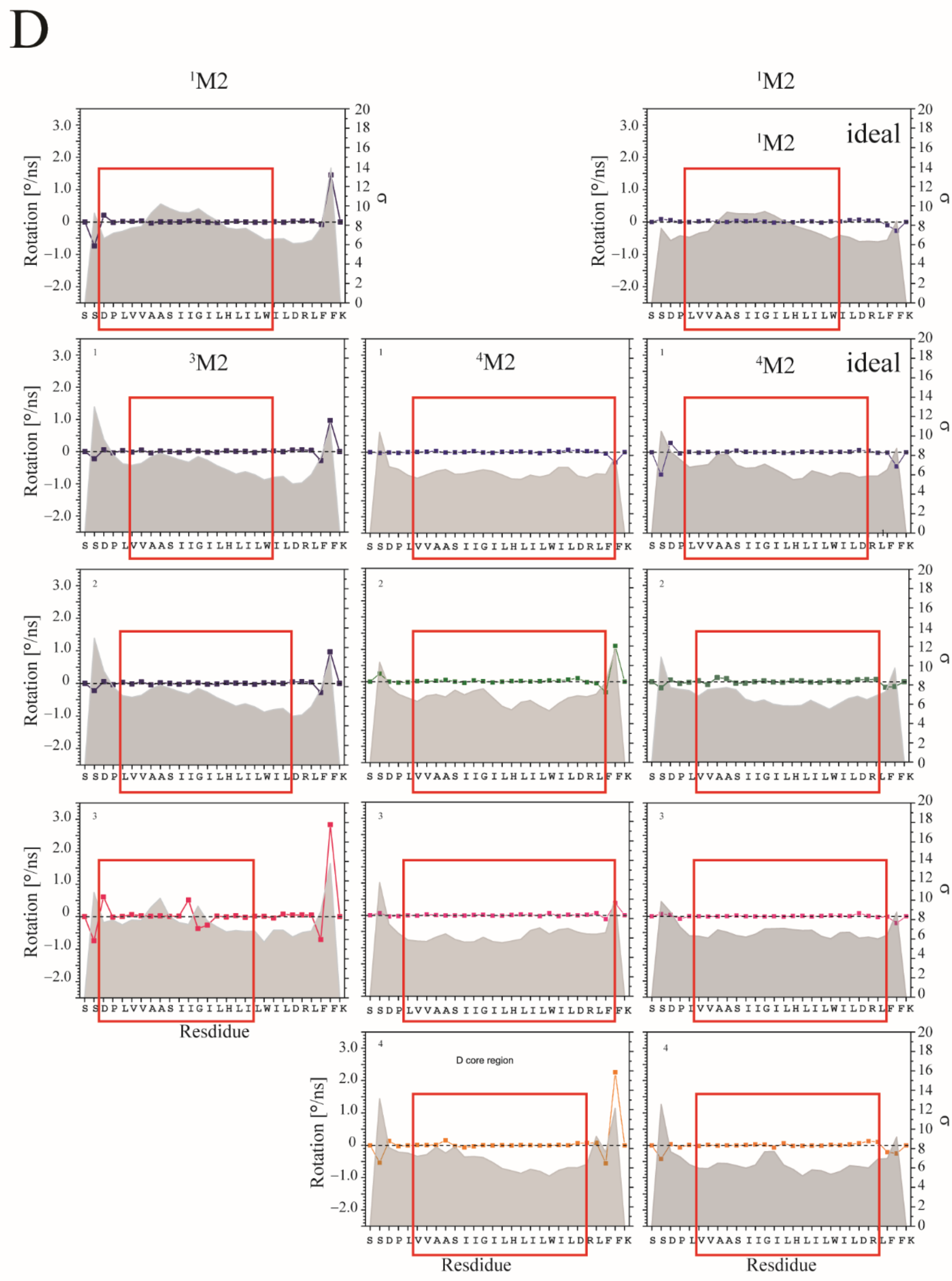
D
[1]M2
[1]M2
ideal
[3]M2
[4]M2
[4]M2
ideal
Rotation [°/ns]
σ
S S D P L V V A A S I I G I L H L I L W I L D R L F F K
Resdidue
D core region

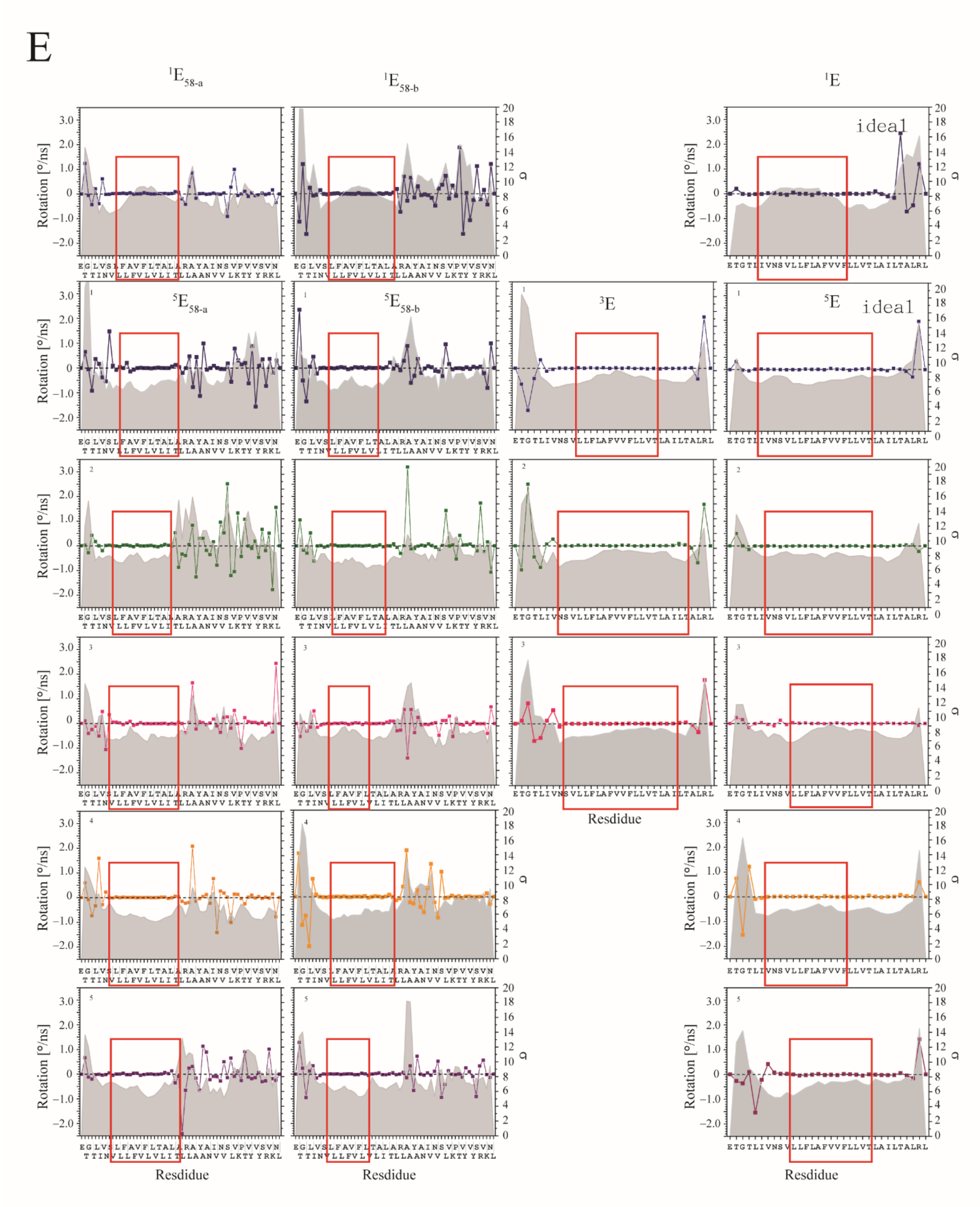
E
1E58-a
1E58-b
1E
ideal
5E58-a
5E58-b
3E
5E
ideal
Rotation [°/ns]
Resdidue
Ω

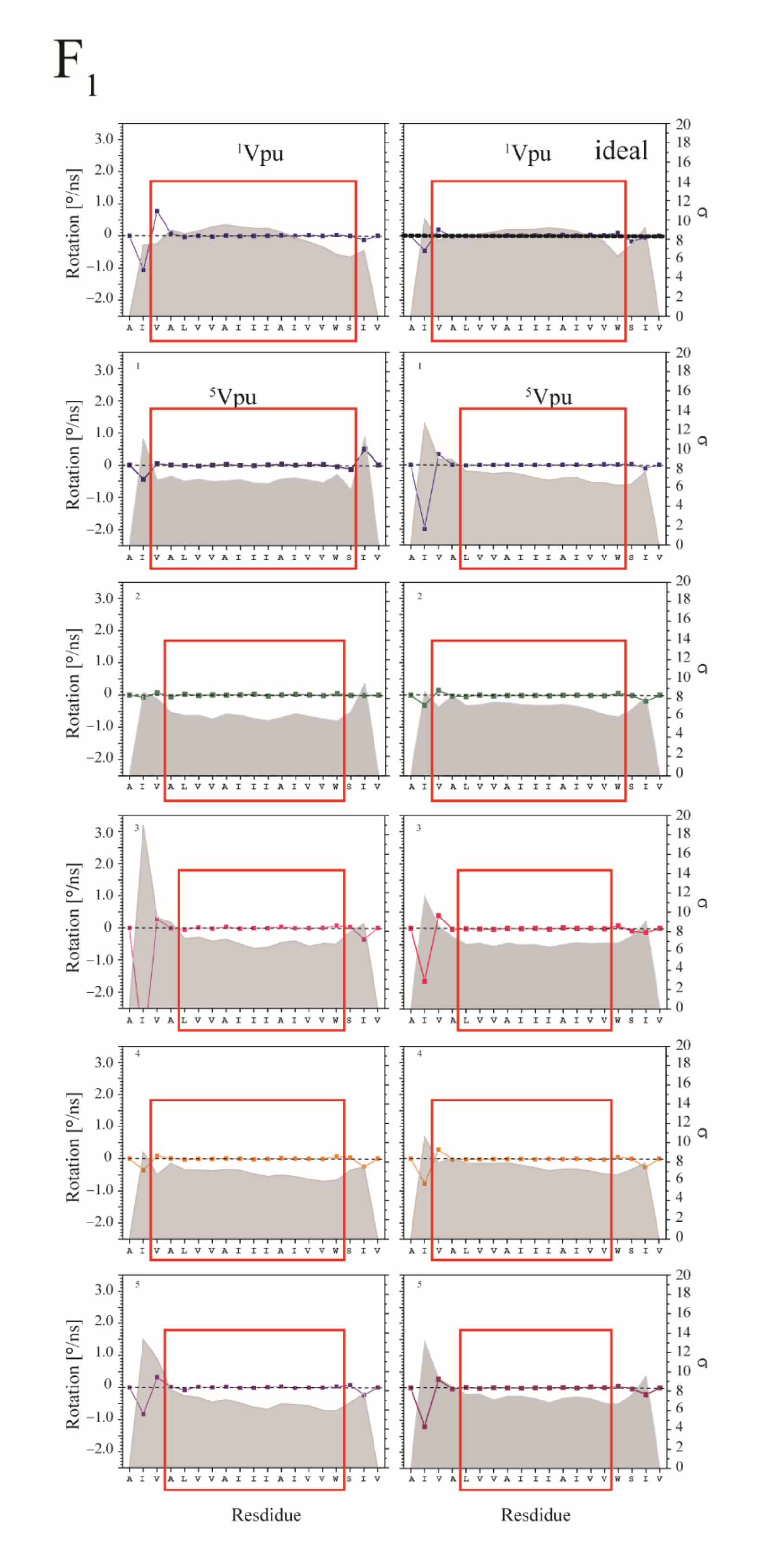

F1
1Vpu
1Vpu ideal
5Vpu
5Vpu
Rotation [°/ns]
σ
A I V A L V V A I I I A I V V W S I V
Resdidue
Resdidue

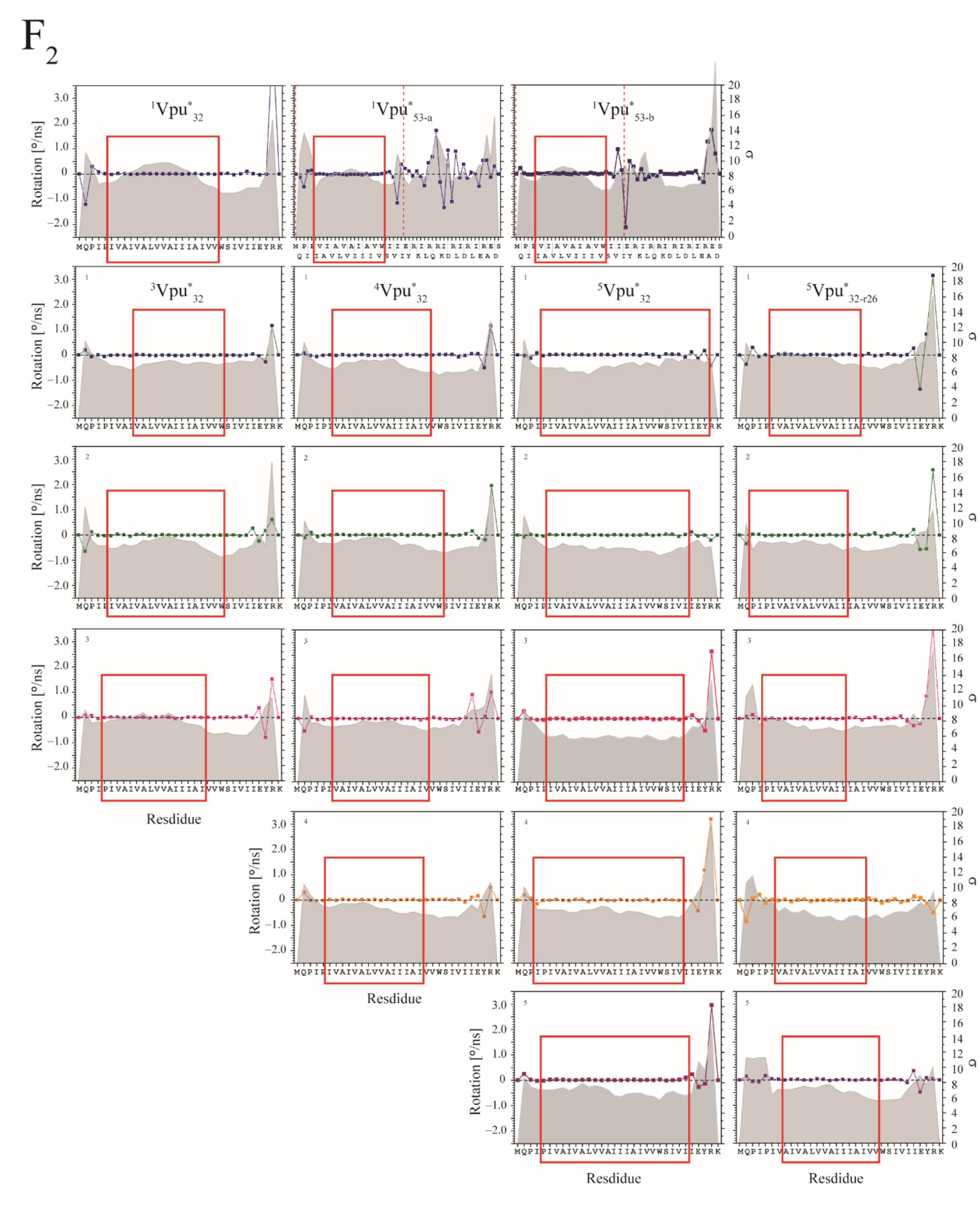

Figure S5. (**A**) Degree of rotation of the Cα atoms per time step (ns) during the MD simulation for each of the residues of the (A) experimental and (**B**) ideal fusion peptides HA, gp41, R696L, and gB as monomers, and also for each TMD in the bundle simulation. (**C**) Degree of rotation of the Cα atoms per time step (ns) during the MD simulation for each of the residues of the ideal fusion peptide S, as monomer and also for each TMD in the bundle simulation. (**D**) Degree of rotation of the Cα atoms per time step (ns) during the MD simulation for each of the residues of the experimental channel proteins ^{1}M2, ^{3}M2 (artificial conformation), ^{4}M2, and the ideal channel proteins ^{1}M2 and ^{4}M2. (**E**) Degree of rotation of the Cα atoms per time step (ns) during the MD simulation for each of the residues of the experimental E proteins as extended peptides $^{1}E_{58\text{-a}}$, $^{1}E_{58\text{-b}}$, $^{5}E_{58\text{-a}}$ and $^{5}E_{58\text{-b}}$, as well as the artificial conformation ^{3}E, which is generated by using experimental monomers as building

blocks, and ideal peptides ^{1}E, and ^{5}E. For the experimental ^{1}E, see Figure 5. (**F$_1$**) Degree of rotation of the Cα atoms per time step (ns) during the MD simulation for each of the residues of the experimental channel protein ^{1}Vpu (left column) and respective ideal protein (right column), as well as (**F$_2$**) the ideal channel proteins $^{1}Vpu^{*}_{32}$, $^{3}Vpu^{*}_{32}$, $^{4}Vpu^{*}_{32}$, $^{5}Vpu^{*}_{32}$, $^{5}Vpu^{*}_{32\text{-}r26}$, and $^{1}Vpu_{53\text{-}a/b}$. 'r26' marks the pentameric bundle in which the tryptophan residues are pointing outside the bundle leaving the S24 pointing inside the putative pore. The standard deviation is shown as grey line. The red boxes mark the amino acids used for calculating the curvature from the respective values of the standard deviations.
